# Supplementary material for: Early Oldowan technology thrived during Pliocene environmental change in the Turkana Basin, Kenya
Source: Nat Commun. 2025 Nov 4;16:9401. doi: 10.1038/s41467-025-64244-x (PMC12586688; doi:10.1038/s41467-025-64244-x)
Supplement: Supplementary file 1 — Supplementary Information [file 41467_2025_64244_MOESM1_ESM.pdf]

Supplementary Material

Supplementary Note 1

Magnetostratigraphy

Paleomagnetic fieldwork and sampling

Paleomagnetic samples were collected during several fieldwork sessions. The initial samples (42 samples), gathered in 2012 from two sections (C and F), showed normal magnetic polarities in section C (lower stratigraphic position), see Supplementary Fig. S03. In contrast, section F (upper stratigraphic position) exhibited reversed polarities, except for a single sample in the middle of paleosol level P4a, which showed normal polarity but was insufficient to indicate a normal polarity reliable interval. In 2017, two new sections (A and B) were sampled, and section C was extended (24 samples). Both sections A and B exhibited normal magnetic polarities, while the extended section C contained a reversed polarity interval at the uppermost sampled level. In 2018, sampling resumed to increase the resolution of sections A, B, and C, and a new section (E) was sampled throughout sites NMT3 and NMT2 (48 samples). Section E included test samples corresponding to levels P3 and C4 collected in 2017 and was characterized by reversed polarities. The remainder of the samples were collected in 2019 (35 samples) and 2022 (11 samples). Section D, developed in 2019, displayed a clear polarity reversal from normal at the base to reversed at the top, within a homogeneous lithologic unit (paleosol level P1). Section G, developed in 2022, provided the uppermost interval of the stratigraphic sequence investigated, exhibited a short (~9m) normal polarity interval at the base, while the top of the section was reversed.

A total of 160 samples collected in the field were measured and interpreted, with the exception of a few that broke apart during preparation. These 160 samples correspond to 147 distinct stratigraphic levels, along with 13 duplicates collected from crumbly levels with poor preservation. Of these, two duplicates were destroyed during measurements before the completion of the demagnetization process. Samples that were damaged during preparation or measurement have been excluded from the analysis and discussion but are reflected in gaps within the sequential numbering system.

Rock magnetism

A series of rock magnetic experiments were conducted to identify the magnetic carrier, to test its reliability in carrying a characteristic remanent magnetization (ChRM), and to gain insight into changes in the magnetic fabric of the samples.

Magnetic Susceptibility Measurements: In the field was used a ZH-SM30 portable magnetic susceptibility meter. Magnetic susceptibility measurements were performed at 130 levels, with five measurements taken and averaged for each level. In cases of instability, the procedure was repeated. To ensure accuracy, susceptibility was not measured in windy conditions. Once in the lab, the samples were measured again using the same meter, and similar values to those obtained in the field were recorded. The results (Fig. 6) show significant changes in magnetic susceptibility.

Subsequently, the rock magnetic experiments were performed using a Lake Shore 3900 Vibrating-Sample Magnetometer (VSM). These experiments included Hysteresis, Isothermal Remanent Magnetization (IRM) Acquisition, and First-Order Reversal Curve (FORC) measurements (Supplementary Data 1, 2, Supplementary Fig. 18).

Hysteresis Measurements: Hysteresis loops were measured to determine the magnetic properties of the samples, such as coercivity, saturation magnetization, and remanent magnetization. By applying a varying magnetic field, the magnetization of the samples was observed, allowing the identification of the magnetic minerals present and their

behavior under different magnetic field strengths. The hysteresis loops were obtained in a magnetic field up to 1T, with averaging time of 200 ms, and field increment of 8 mT. The slope-corrected hysteresis loops were mass normalized to obtain the parameters of saturation magnetization (Ms), remanent magnetization (Mrs) and the coercivity (Hc).

Isothermal Remanent Magnetization (IRM) Acquisition: IRM acquisition experiments were conducted to assess the ability of the samples to acquire and retain a remanent magnetization when exposed to a series of increasing magnetic fields. The IRM was measured up to 1.5 T in 150 points with an averaging time of 100 ms.

First-Order Reversal Curve (FORC) Measurements: FORC measurements were performed to characterize the magnetic interactions and domain states within the samples. By analyzing a series of partial hysteresis loops, the FORC diagrams provided detailed information about the magnetic grain size distribution, interaction fields, and the presence of single-domain, multi-domain, or pseudo-single-domain particles. A total of 280 FORCs were measured for samples P1 and P5 to characterize the distinct signature from hysteresis loops, with the horizontal profile along the Bc between 0 and 150 mT, and the vertical profile (Bu) between -50 and 50 mT. The maximum applied field was 1T, and the averaging time of 400 ms. FORCinel software[1], with the VARIFORC algorithm of Egli [2] was used for smoothing (Sc0 = 7, Sb0 = 3, Sc1 = Sb1 = 7, horizontal and vertical lambda of 0.1), which provides smaller smoothing in regions near Bu = 0 and larger smoothing for other regions.

DCD Demagnetization: A series of Direct Current Demagnetization (DCD) experiments were also conducted to further evaluate the stability and coercivity spectra of the remanent magnetization. DCD involves applying a reverse magnetic field to progressively demagnetize the samples. This method helps in understanding the coercivity distribution and the stability of different magnetic components within the samples. The DCD results were combined with the Hysteresis data for the Day Plot Analysis.

Day Plot Analysis: Additionally, the results from the hysteresis and remanent coercivity (Hcr) were used to create Day plots (Supplementary Fig. 18). These plots enabled the classification of the magnetic domain state of the samples by comparing the ratios of remanent to saturation magnetization and remanent coercivity to coercivity. The Day plot provided a visual representation of the magnetic domain states of the magnetic minerals.

The rock magnetism data (Supplementary Data 1, Fig. 18) suggest a low coercivity magnetic component signature for most of the samples, probably magnetite. P1 paleosol sample shows a distinguished wasp-waisted hysteresis loop indicative of a mixed low and high coercivity magnetic minerals composition; this is also evident in the IRM curve, which shows a high coercivity component not fully saturated at 1.5T. Pseudo-single domain (PSD) appears to be the main domain state from the Day Plot. P1 sample plot distinctly compared to the other samples in the Day plot, most likely due to the very high remanent coercivity component (e.g., hematite) of the sample. FORC diagrams of samples P1 and P5 appear to have a similar signal, with few distinct signatures. While P1 shows a wider vertical interaction, a characteristic of coarse grain multidomain (MD) particles, P5 shows a stronger signal near the central ridge indicating PSD behavior. The variation in the magnetic signal between sample P1 and the other samples is possibly due to the presence of a higher coercivity component of mineral hematite, which may have formed from enhanced oxidation during the pedogenesis. P1 paleosol is a vertisol, which generally develops under increased seasonal wetting and drying cycles. Low MAP estimate for P1 paleosol indicates increased period of dry season under arid climate, which led to enhanced production of hematite from precursory minerals, as was also seen in paleosols of other regions of the Turkana basin[3].

Magnetostratigraphy

We used progressive thermal demagnetization and/or progressive alternating field demagnetization to isolate the ChRM. The natural remanent magnetization (NRM) was thermally demagnetized and measured using a 2G

Enterprises DC Superconducting Quantum Interference Device (SQUID) cryogenic magnetometer, which has a noise level of 3 × 10^-12 Am^2. Heating was performed in a laboratory-built, magnetically shielded furnace with a residual field of less than 10 nT. Additionally, alternating field demagnetization was performed in small increments up to a maximum of 100 mT using an in-house robotized sample handler attached to a horizontal 2G Enterprises DC SQUID cryogenic magnetometer [4].

Thermal and alternating field demagnetization of Namorotukunan samples revealed a weak, low-temperature (LT), viscous overprint that was generally removed at 150°C and 15 mT (Supplementary Fig. 18). A second higher temperature (HT) component, which we consider corresponding to the ChRM, was demagnetized at temperatures between 150 and 600°C or fields between 15 and 45 mT. This component was averaged from four or more consecutive temperature/field steps and calculated using principal component analysis. We obtained ChRM directions for 135 samples of both reversed and normal polarities (Supplementary Data 1, Supplementary Figs. 3 and 18).

For plots and statistical analysis, we used only one sample per level, discarding duplicates with higher mean angular deviation (MAD). The selected samples were assigned to four qualitative groups. The highest quality directions, represented by samples with MAD values below 7°, were attributed to group G1 (133 specimens). A second group, G2, representing average quality directions with MAD values between 7° and 17°, were plotted as circles and used in the polarity interpretation (Supplementary Fig. 16). A third group, G3, contains 12 specimens with poor quality directions and were plotted as stars in Supplementary Fig. 16 but were not used for the polarity interpretation. A fourth group, X, contains specimens that did not show consistent directions and were not plotted.

The final polarity pattern of the Namorotukunan section consists of four intervals: two of normal polarity and two of reversed polarity (Fig. 3 and Supplementary Figs. 3 and 18). The lowermost part of the section, between 0 and ~16.5 m, comprises a long normal polarity interval (NZ1) that we correlate to the normal polarity chron C2An (Gauss) of the GPTS (Fig. 3 and Supplementary Fig. 3), placing the oldest artifact site well within the upper normal interval (C2An.1n) of the Gauss Chron. The reversed polarity interval, RZ1, between ~16.5 and ~36 m, correlates to C2r. We detected a short normal polarity zone, NZ2, between ~36 and ~45 m, which correlates to the C2r.1n (Feni) magnetic excursion. Above this interval, we identified a second reversed polarity interval, RZ2, above 45 m, corresponding to chron C2r.1r. This correlation implies that the oldest artifact level (NMT1) in Namorotukunan is located in the Gauss chron, while younger artifact levels (NMT2 and NMT3) belong to the Matuyama chron. Combining the magnetostratigraphic markers, the tephra marker, and the paleoenvironmental change marker allows us to develop an age model for the studied section (Fig. 3).

Supplementary Note 2:

Chemical index of alteration (CIA) and Mean annual precipitation (MAP) using paleosols chemistry

Chemical index of alteration (CIA)

The CIA, first introduced by Nesbitt and Young[5], is a measure of intensity of chemical weathering of parent or source rocks often employed to paleosols and sediments to understand climate conditions as temperature and precipitation are major driving forces for the chemical weathering. CIA is calculated as = 100 × (Al_2O_3/Al_2O_3+CaO*+Na_2O+K_2O)][5]. Here major oxides concentrations are first converted into moles. CaO* represents concentration of CaO mole in silicate fraction of sediments and a correction for carbonate (calcite and dolomite) and apatite is applied [5,6].

The carbonate and apatite corrections for CaO are normally accomplished by calculating corrections from measured CO_2 and P_2O_5 mole contents[6]. In the absence of CO_2 mole data, approximate corrections can be made by assuming reasonable Ca/Na ratios in silicate material [7]. After apatite corrections, if the remaining number of CaO moles is less

than that of Na_2O, this CaO value is adopted. If the number of moles is greater than Na_2O, CaO* was assumed to be equivalent to Na_2O [7]. Since Ca is typically lost more rapidly than Na during weathering, it will give minimum (by up to about 3 units) CIA values[8]. To avoid or minimize the under-estimation of CIA values, several paleosol samples having CaO wt% higher than >5%, were reanalyzed after pedogenic carbonate removal using 1N HCl treatment for the estimation of CaO* in silicate fractions.

As chemical weathering increases, more labile cations (e.g., Ca^{+2}, K^+ and Na^+) are leached from primary minerals (e.g., feldspar) of parent rocks and stable residual elements (e.g., Al^{+3}) are retained forming secondary clays [5,6,9]. Therefore, higher CIA values indicate higher chemical weathering and vice-versa. CIA for paleosols ranges from 51 to 64 indicating incipient to moderate weathering of paleosols.

Mean annual precipitation (MAP)

Several climofunctions have been developed for the reconstruction of the MAP using the geochemistry of paleosols. MAP for all paleosols (except for P2b and P1) was calculated using the following empirical equation proposed by Sheldon and colleagues[10].

$$\text{MAP (mm yr}^{-1}\text{)} = 221.1e^{0.0197(\text{CIA-K})}$$

Where the standard SE is ±181 mm yr^{-1}. CIA-K was calculated using the following equation of Maynard[11] based on the whole rock geochemistry of paleosols.

$$\text{CIA-K} = 100 \times (Al_2O_3)/(Al_2O_3+CaO+Na_2O)$$

The MAP using this empirical equation has been universally applied to paleosols of various types and ages, except for paleosols with near surface carbonates or evaporite minerals, for hillslope or montane settings, or for laterites [9,10].

The P2b and P1 paleosols are vertisols having dominant slickenside signatures. CALMAG based MAP proxy (named here as MAP_{CM}), developed exclusively for the vertisols, provides a better estimate of precipitation compared to CIA-K [12].

$$\text{CALMG} = 100^* (Al_2O_3)/(Al_2O_3+CaO+MgO)$$

$$MAP_{CM} = 22.69 \times \text{CALMAG} - 435.8$$

The SE for MAP_{CM} is ±108 mm yr^{-1}. CaO values for P2b and P1 are ~6.98 and 9.29%, respectively, and are within threshold (CaO <10%) for CALMAG proxy [12].

The MAP for paleosols varies between 231 and 855 mm yr^{-1} broadly classifying climate as semi-arid to semi-humid. The MAP estimates using CIA-K have been suggested to be useful for semi-arid or arid regions [9]. However, recent studies recommend a ≤5% CaO threshold for MAP using CIA-K relationships[13,14]. Dzombak and colleagues [13] suggested that CaO >5% in paleosols might underestimate MAP using CIA-K, but can be used in understanding relative changes in precipitation. CaO in paleosols ranges from ~1.85 to 9.73% (avg. 4.66%), except for paleosols P0 and R4 which have CaO ~21 and 25.5%, respectively. The very high CaO in both the paleosols are due to high pedogenic carbonates ($CaCO_3$ = 34% for P0 and 43% for R4). While MAP for both the P0 and R4 paleosols ~309 and 294 mm yr^{-1}, respectively, are under-estimated, but nonetheless these paleosols show arid climatic conditions.

The precipitation range during the studied time interval is consistent with the findings of Fortelius and colleagues [15] who used an ecometric model for MAP estimates in the Turkana basin. The other independent estimates on MAP from the East Turkana basin during the studied time interval are also in accordance with present MAP results [16,17].

Supplementary Figures

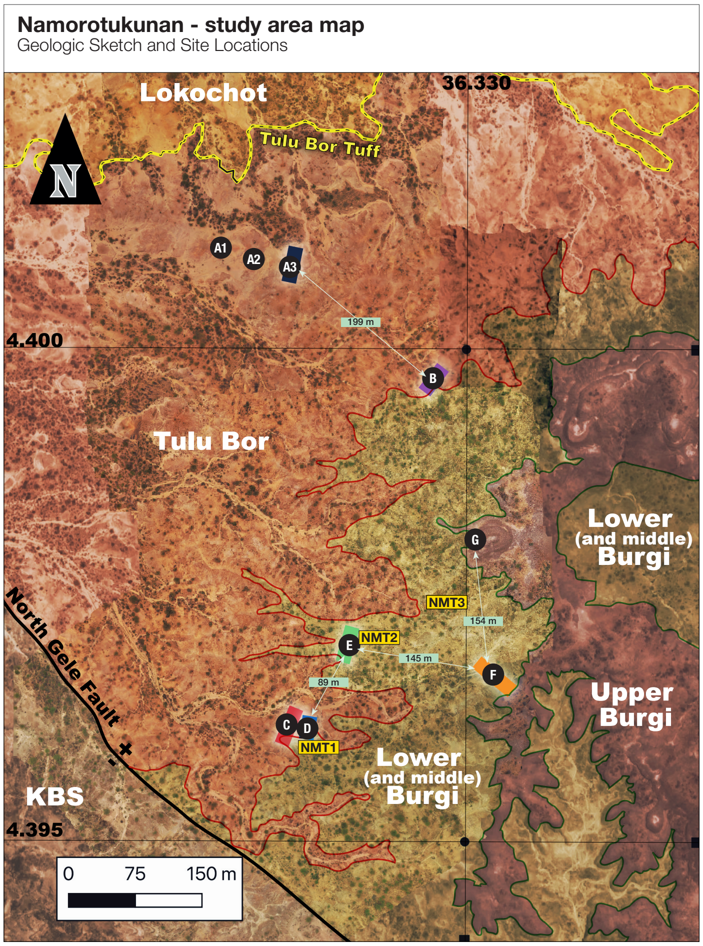

Supplementary Fig. 1 | Aerial Composite of the Study Area with Geological Annotations. This figure presents a composite aerial photograph of the study area, superimposed with a simplified geological sketch. Key features include: the yellow line indicating the tephra layer associated with the Tulu-Bor tuff, marked further by solid and dashed black lines to show the observed and extended areas, respectively, a red line delineating the base of a geological unit characterized by alternating layers of gravels, floodplain silts, and paleosols, a green line tracing the base of the lake clays and a prominent black line representing the North Gele Fault, a significant normal fault evident throughout the region. The map provides exact locations of stratigraphic sections (marked from A to F) investigated in Area 40.

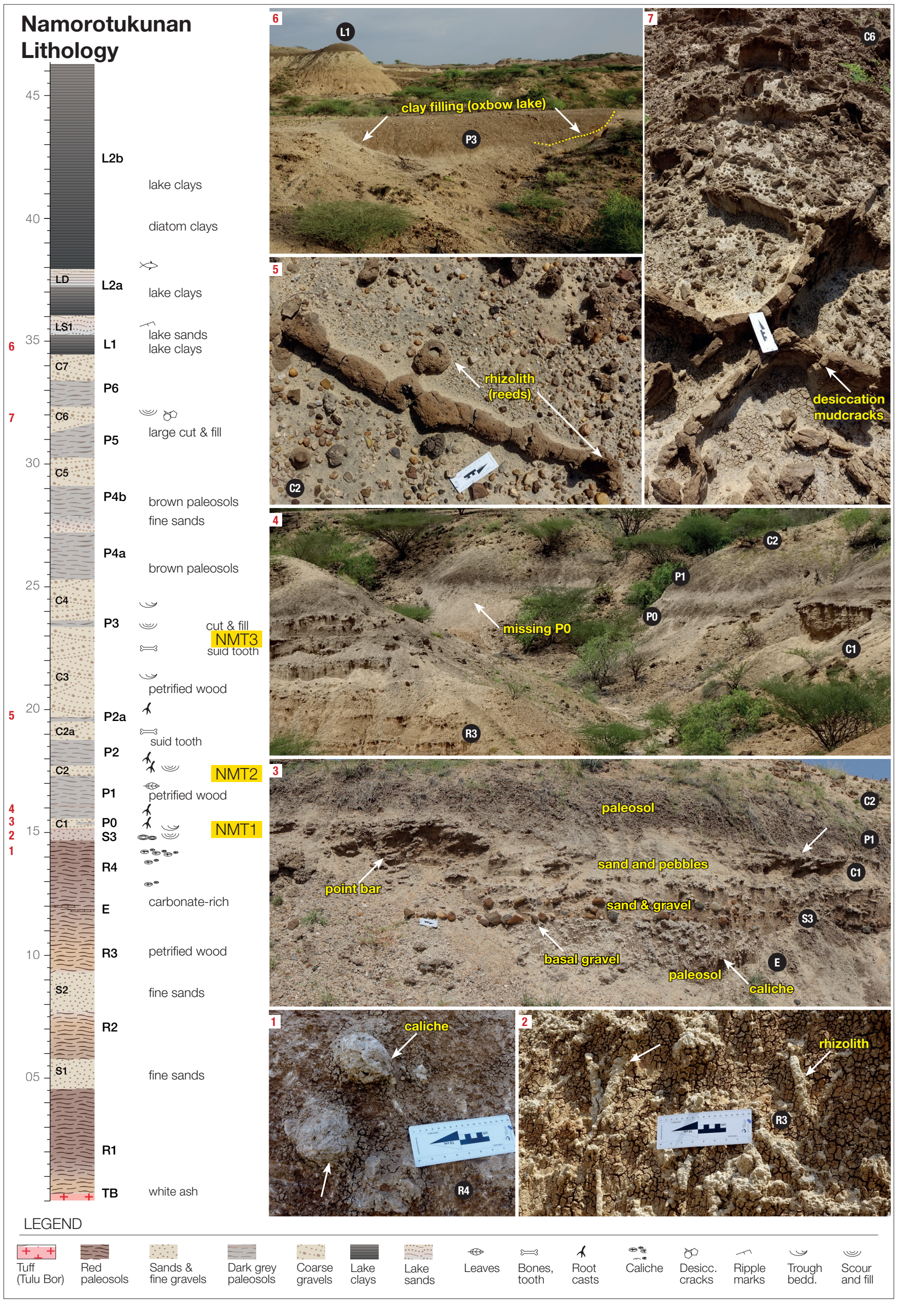

Supplementary Fig. 2 | Synthetic Lithological Column of the Studied Section. The main lithologies include red paleosols (R1–R4), sands and poorly cemented sandstones (S1–S3), grey to grey-brown paleosols (P1–P6), conglomerates and gravels (C1–C7), lacustrine clays (L1–L2b), lacustrine sands with ripple marks (LS1), and diatomite-rich lacustrine clays (LD). These lithologies reflect a variety of depositional environments, from fluvial and alluvial systems to lacustrine settings. The images next to the figure illustrate the specific features observed identified in the studied section:1. Carbonate concretions; 2. Rhizoliths at two different levels, 3. Paleo-channel fills featuring gravel point bars interspersed with floodplain deposits and paleosols, 4. Discontinuous levels of floodplain paleosols, 5. Additional rhizoliths, 6. Oxbow lake clay deposits, 7. Desiccation cracks

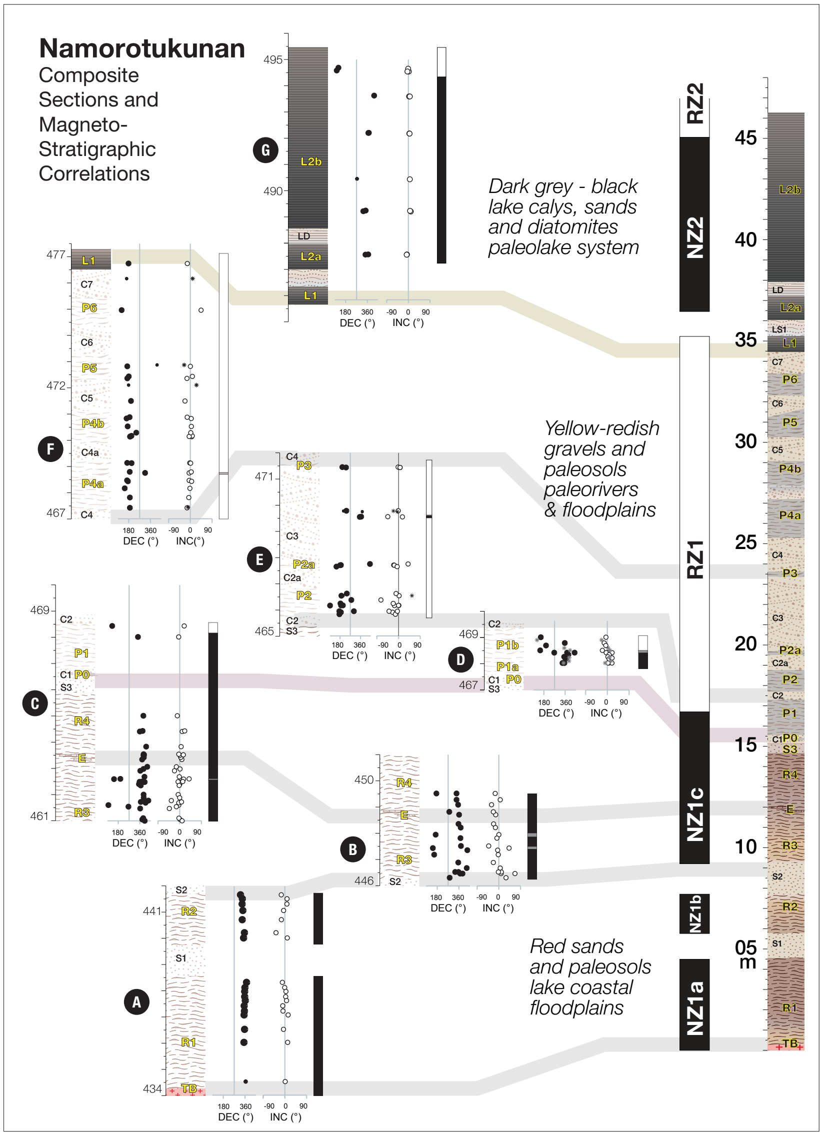

Supplementary Fig. 3 | Magneto-Composite Synthetic Magneto-Lithostratigraphic Column with Stratigraphic Correlations. This diagram presents the magneto-stratigraphic results and correlations across a synthetic magneto-lithostratigraphic column. The column is divided into seven sections (A-G), each geologically characterized and sampled for paleomagnetic analysis and paleoenvironmental proxies. The magnetostratigraphic results (declination, inclination and inferred magnetic polarity) are plotted along the lithological columns of each studied site. We combined the litho-stratigraphic observations from sections A-G to produce a composite, synthetic stratigraphic column (to the right side of the figure). The sections collectively span most of the composite stratigraphic sequence and feature: 1. Two reverse polarity intervals labeled as RZ1 and RZ2. 2. Two normal polarity intervals, NZ1 and NZ2. Notably, the normal polarity interval NZ1 is discontinuous due to the presence of sandy units, and it is subdivided into three sub-intervals (NZ1a, NZ1b, NZ1c). We also describe three magnetic polarity reversals (NZ1c/RZ1, RZ1/NZ2, NZ2/RZ2).

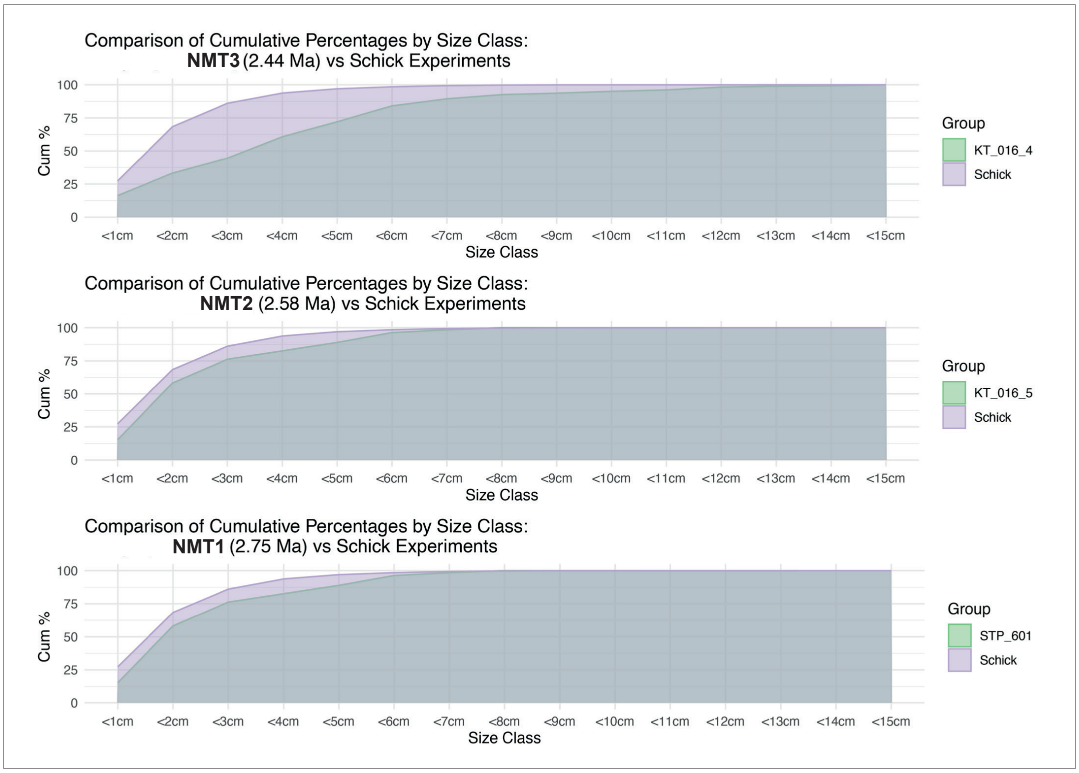

Supplementary Fig. 4 | Size-class analysis. The cumulative distribution of the maximum dimension of all artifacts from the three archaeological sites. The distribution is compared to the experimentally produced assemblages of Schick[18]. The close association between the experimentally produced assemblage and the cumulative distribution of the archaeological assemblages indicates relatively minimal size sorting. Lower frequencies of smaller objects in the 2.44 Ma assemblage (NMT3) indicate some winnowing. This is also associated with higher degrees of artifact orientation suggesting higher energy contexts for this locality.

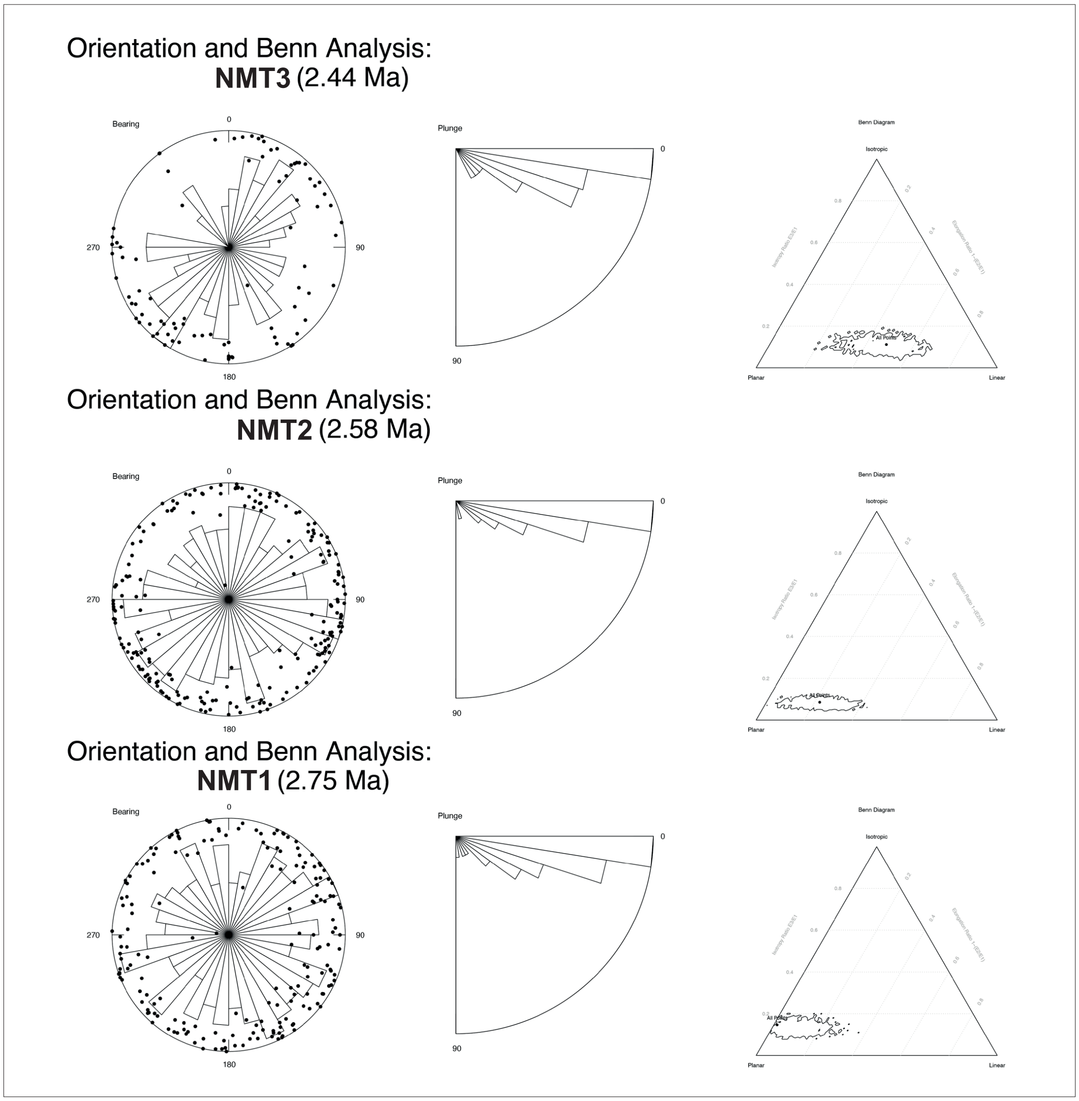

Supplementary Fig. 5 | Orientation analysis. Fabric analysis of the artifacts from the three archaeological horizons. Analysis of oriented specimens in each of the three excavations. On the left, Schmidt lower hemisphere plots summarize the bearing and plunge angles with a superimposed Rose diagram summarizing only the bearing angle distributions[19]. These plots indicate the lack of oriented specimens in the 2 older units. The middle figure represents a circular histogram of plunge angles. The lack of numerous steeply angled specimens indicates most artifacts were laid down on a flat surface[20]. Right hand plots represent Benn statistics computed on 40 closest artifacts (using a resampling procedure to provide generalized patterns of orientation). These ternary plots indicate the general trends of orientation and plunge showing a planar (non-oriented but laid down on a flat surface) pattern for the two oldest assemblages. The more linear pattern in the youngest assemblage suggests some of the artifacts may have been oriented by river action.

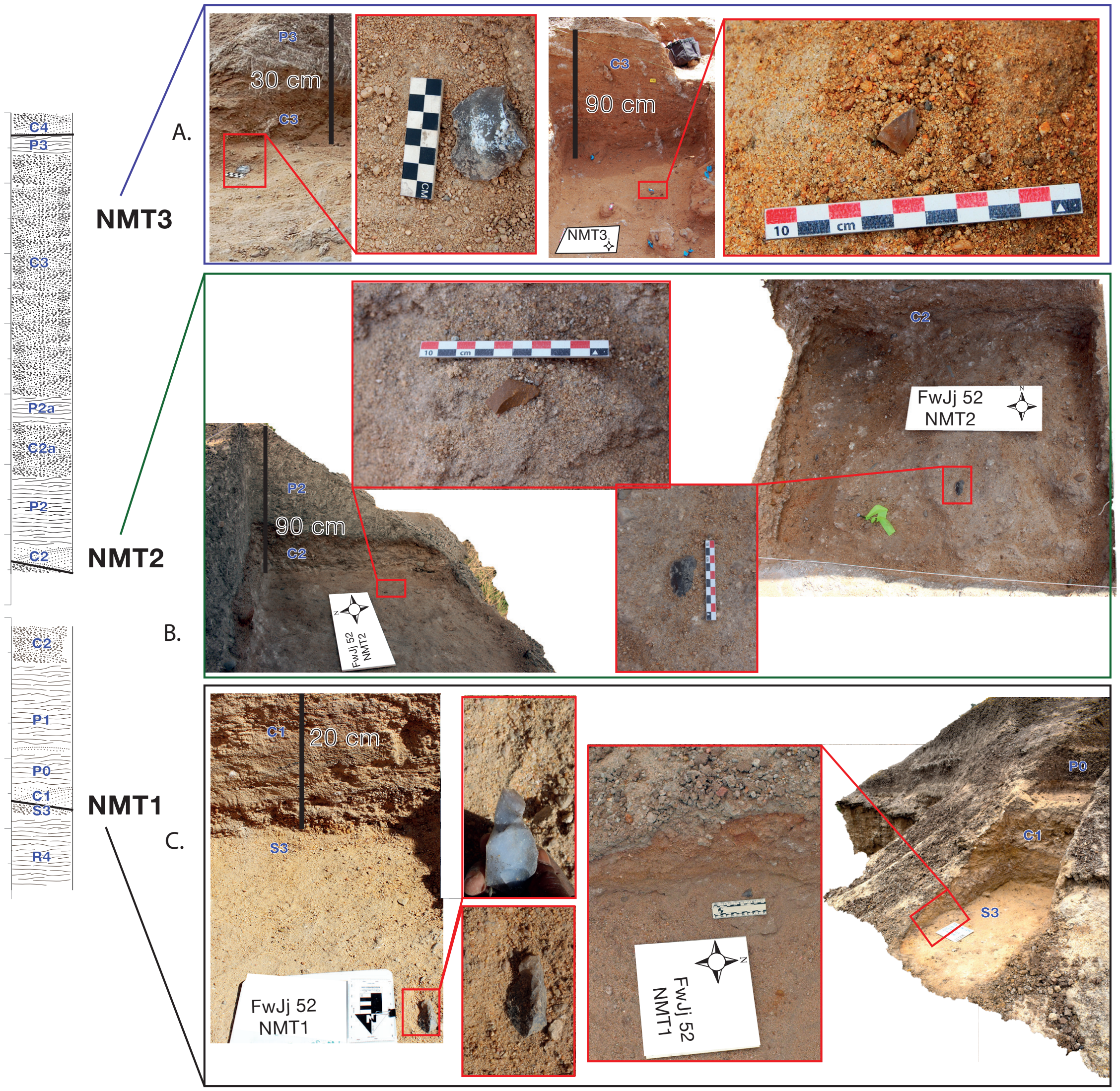

Supplementary Fig. 6 | *In situ* photos. A. Artifacts recovered from the excavation of NMT3. A large section of cross bedded sands (stratigraphic unit C3) incorporates the stone artifact as well as numerous cobbles associated with a braided river systems. B. The excavation at NMT2 recovered numerous artifacts in the base of a paleosol unit (stratigraphic unit P0) as well as the underlying sands and gravels (stratigraphic unit C2. C. The excavations at NMT1 record the presence of stone artifacts in a conglomeratic unit (C1) as well as the underlying sandy unit (S3).

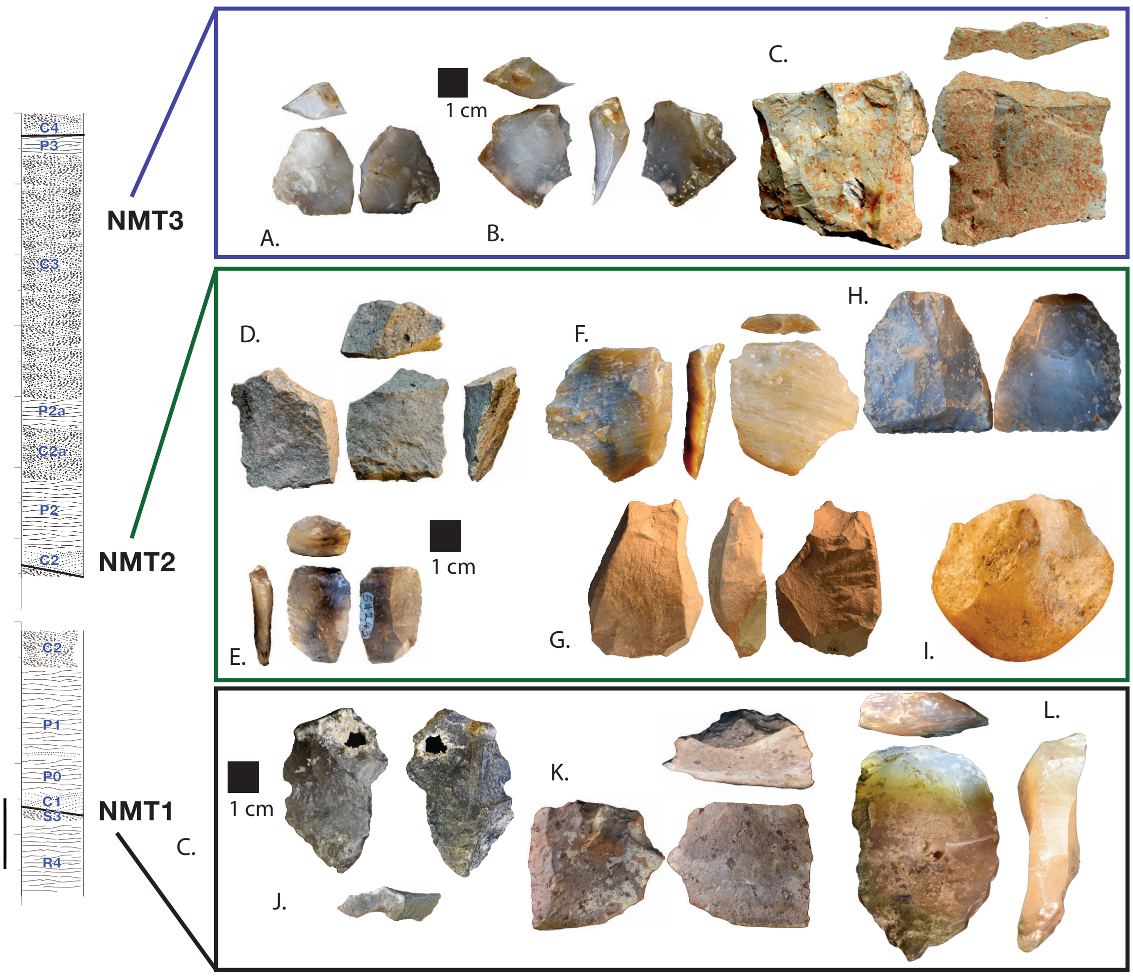

Supplementary Fig. 7 | Artifacts. A.-C Artifacts recovered from NMT3. Panels A. and B represent small chalcedony flakes. Panel C. represents an ignimbrite flake. D.-I. Artifacts recovered from the excavation of NMT2. Panel E., F., and H. represent small chalcedony flakes. Panel D. is an ignimbrite flake. Panel G. is an ignimbrite core. Panel I. is a small quartzite core. J.-L. Artifacts recovered from NMT1. Panels J. and L. represent small chalcedony flakes. Panel K. represents a small ignimbrite flake.

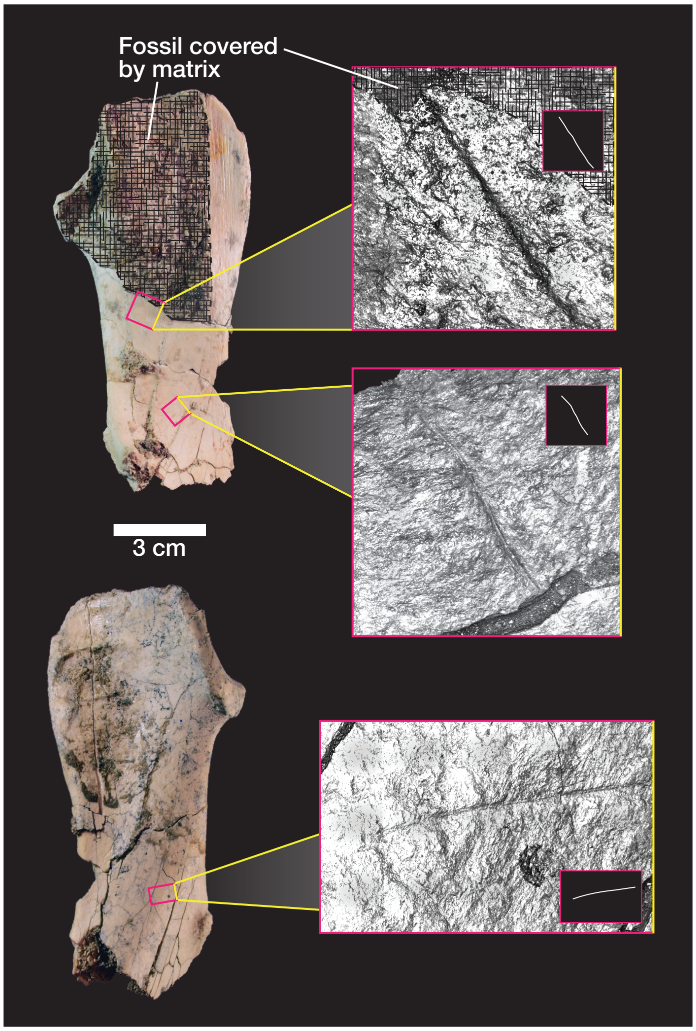

Supplementary Fig. 8 | Bone Surface Modifications: 32 fossil bones were recovered from *in situ* excavations around Namorotukunan. A small proportion of these fossils (4 bones) have well-preserved surfaces that record evidence of butchery and carnivore damage. Too few specimens are available to make any meaningful interpretation about the sequence of access to carcasses. The fossil bone displayed here is a portion of a Size 3 suid innominate. The fossil is partially covered in a matrix of carbonate that was impossible to remove without damage. Other fossils with potential hominin damage include 3 separate long bone fragments that cannot be attributed to element based on their morphology. Three separate surface modifications on the suid innominate confirm the use of stone tools to access animal tissues on this carcass. These linear marks have internal striations that are consistent with the morphology usually associated with stone tool cut marks [21,22]. Inset images were collected using a Nanofocus spinning disk confocal microscope and represent 3D surfaces captured to enhance the internal striations in these modifications. As the inset images represent 3D models, a standard scale cannot be accurately placed on these images because they represent the specimen from a particular perspective.

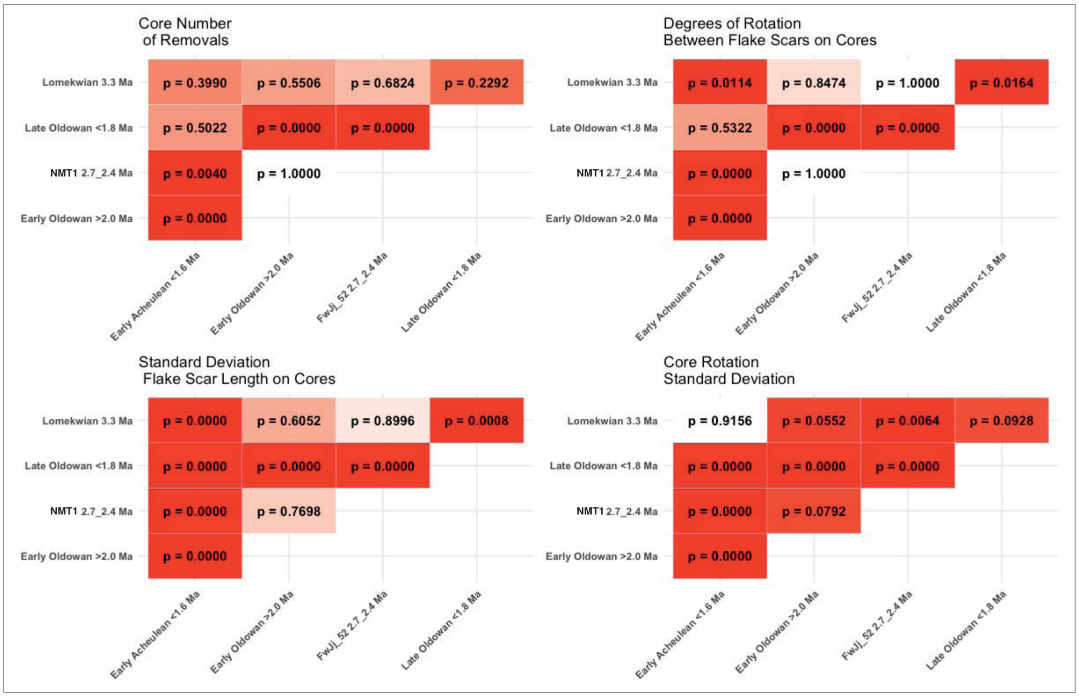

Supplementary Fig. 9 | Statistical Significance of Comparisons with Oldowan Sites. The analysis of technological patterns was carried out on three dimensional models that were captured at the National Museums of Kenya (see Methods). Comparisons of technological patterns between Late Oldowan (n=169), Lomekwian (n=5), and Early Oldowan (n=80) and an assemblage of cores from the NMT1 through NMT 2 assemblages (n=15) indicate that there are significant differences between groups. We used a resampling method to compare the differences (in medians) between groups of assemblages compared to randomly generated sample populations with the same n values from the entire population within this study. Significance is calculated based on the likelihood of finding similar differences in median values within the study group based on random samples of similar n values.

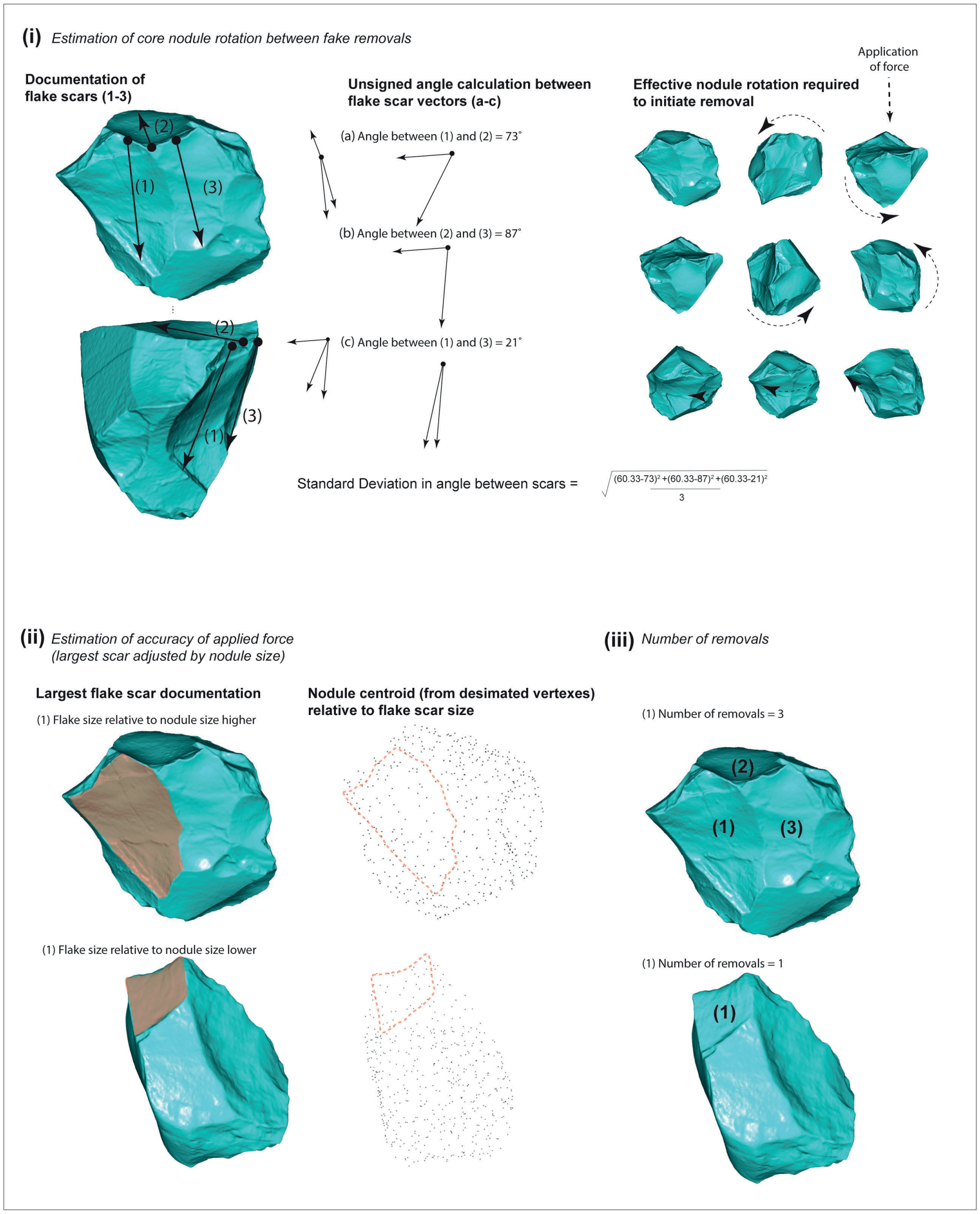

$$\sqrt{\frac{(60.33-73)^2+(60.33-87)^2+(60.33-21)^2}{3}}$$

Supplementary Fig. 10 | Description of Three-Dimensional Analysis. Core forms were scanned from collections at the National Museums of Kenya from the Koobi Fora Formation (FwJj 20; FxJj 10; FxJj 18; FxJj 50; FxJj 1; FxJj 63; FxJj 65) as well as cores from the Kanjera South Formation (KJS1). These samples also include cores from the Ledi Geraru research area which were scanned at the National Museums of Ethiopia (BD 1). (i) Three dimensional models were landmarked to identify the origin and extent of flake scars. These vectors were used to calculate the angles between flake scars and the diversity of angles represented in a single core[23]. Calculations are described in the figure. (ii) Flake scars on cores were characterized by planes on cores that were outlined, and the three-dimensional components of this surface were compared to the overall centroid size of the core. Flake scar count represents the number of removals (larger than 1 cm) on each core.

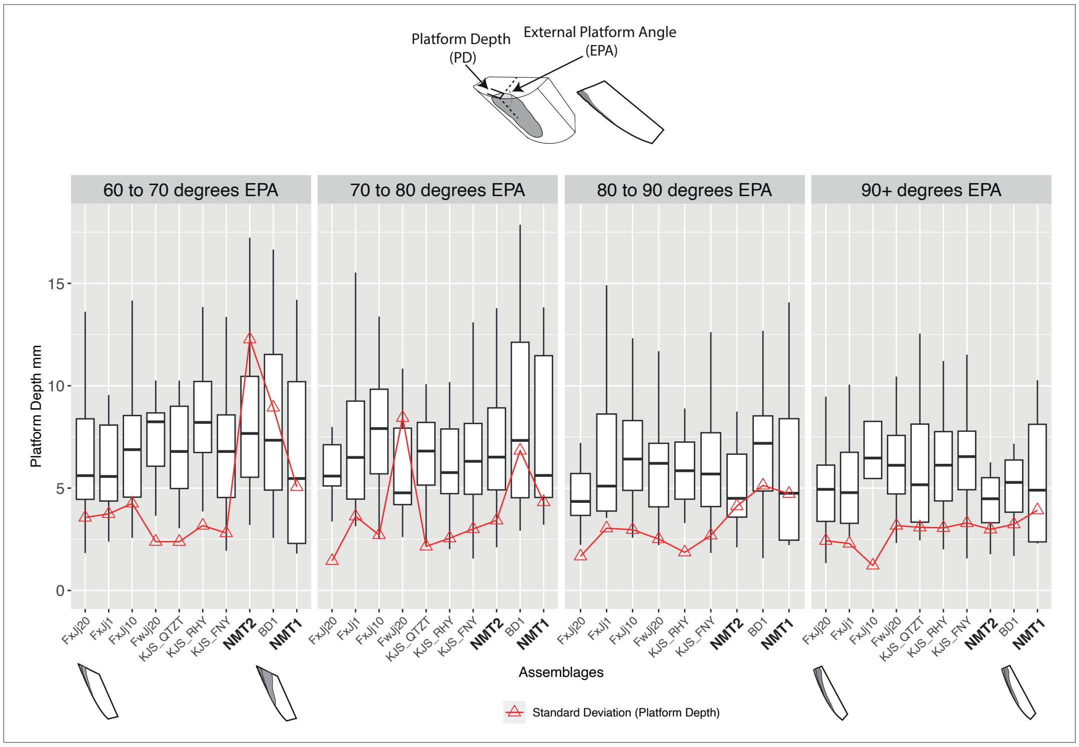

Supplementary Fig. 11 | Technological Decisions: Comparison of variation in platform depth within specific external platform angle intervals for the assemblages from Namorotukunan and several other Oldowan sites [Bokol Dora 1 (2.58 Ma, n=119); Kanjera South (2.0 Ma, QTZT, n=55; RHY,n=94; FNY,n=487); FxJj1-Koobi Fora (1.87 Ma, n=59); FxJj10-Koobi Fora (1.78 Ma, n=169); FxJj20- Koobi Fora (1.5 Ma, n=159); NMT1, n=24; NMT2, n=121]. Date is arranged in age order such that older assemblages are at the right end of each facetted graph. Images of cores and flakes redrawn from Dibble and Rezek[24]. Box plots represent interquartile ranges. Lines outside of box plots represent the first and fourth quartile. Lines within boxplots represent the median value. The red triangles represent the standard deviation within each assemblage. Oldowan assemblages older than 2.0 Ma show significantly greater variation than other Oldowan sites especially in the lower platform angle categories. To assess significant differences in variance between assemblages we used a Monte-Carlo resampling procedure to assess the likely differences between random selections from the entire dataset using similar sample size values. The standard deviation of platform depth values in the Namorotukunan assemblages (within EPA groups) have significantly lower ($p < .05$) standard deviation values than all assemblages younger than 2.0 Ma (with obvious exceptions: FwJj 20: 70 to 80 degrees EPA). Variation in platform depth is substantially lower in high EPA categories (> 90 degrees). At higher external platform angles the amount of force required to initiate fracture increases exponentially with increases in platform depth and likely places physical limits on the variation that can exist in this category[25].

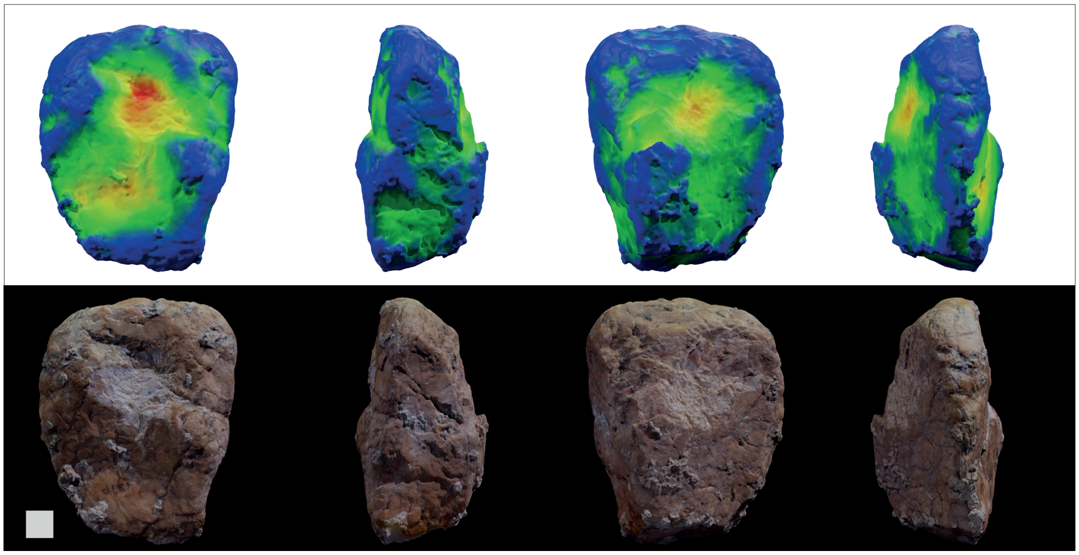

Supplementary Fig. 12 | Percussive tools. There are fewer than three specimens from all three Namorotukunan assemblages classified as percussive tools. Here we use a 3D morphometry[26] to quantify patterns of surficial damage associated with percussive activities. This specimen is a block of feldspar rock that was recovered from the excavation and shows clear evidence of percussive activity on both sides of this specimen. The color ramp on the upper image identifies areas where the surface of the specimen shows an increased depth relative to the rest of the external surface. The color reflects the distance between a convex hull of the surface of the object and the actual vertices associated with the underlying three-dimensional model. Previous reviews have shown that this methodology highlights areas of increased roughness associated with percussive damage patterns. This methodology has been described previously to provide indications of where percussive damage is located on a specimen[26].

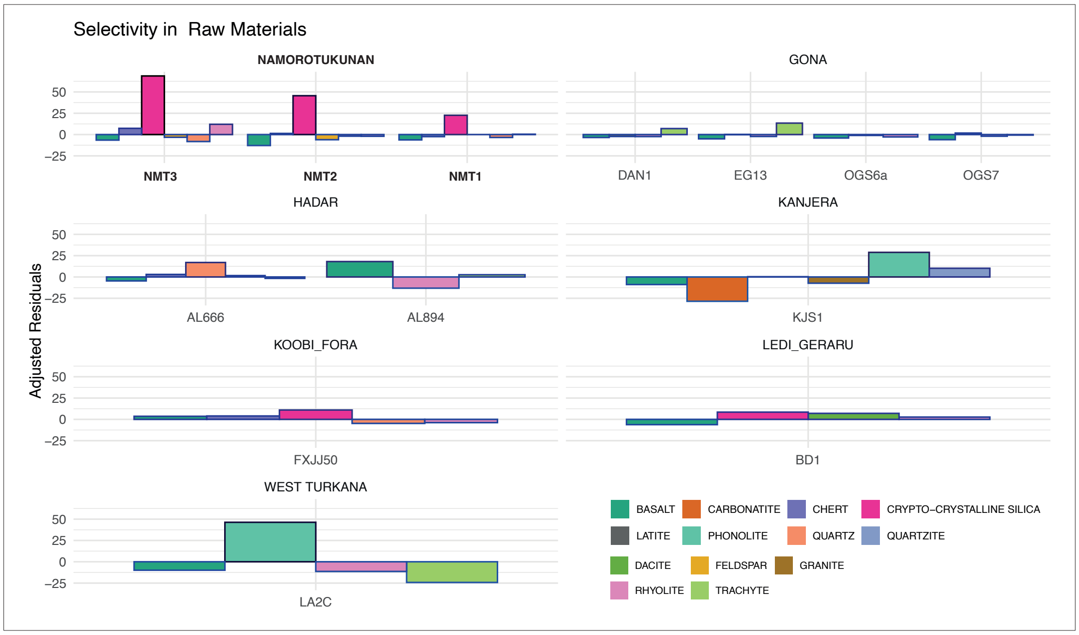

Supplementary Fig. 13 | Selectivity of raw materials. Adjusted residuals for chi-square analysis of rock types found in archaeological assemblages compared to the rock types found in nearby conglomerates. The adjusted residuals indicate the amount of selection relative to the availability of certain rock types. An adjusted residual of 0 indicates that a specific rock type is selected at the same rate as its presence in nearby conglomerates. Higher adjusted residuals indicate higher degrees of selection. Negative adjusted residuals indicate that rocks are underrepresented in the archaeological collection relative to their availability in nearby conglomerates. The very high (>25) adjusted residuals for chalcedony in the Namorotukunan assemblages indicate very high selectivity for these materials. Note similarly high levels of selectivity for phonolite at Lokalalei 2C as previously described by Harmand[27].

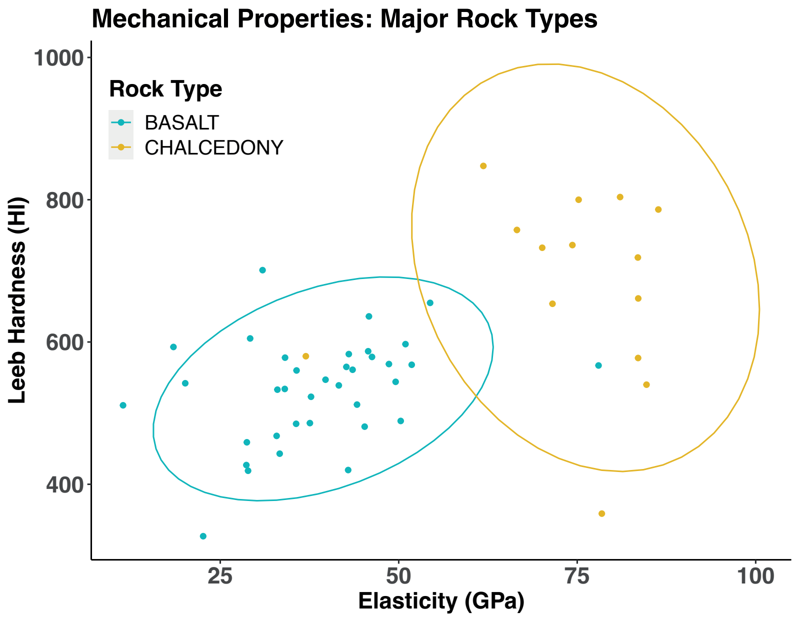

Supplementary Fig. 14 | Mechanical Properties: Rock mechanical properties for the two major rock types in the archaeological assemblages (basalt, chalcedony). Leeb hardness was measured using a Proceq Leeb Hardness tester. The Leeb hardness tester fires an impact body with an indenter made of very high known hardness (carbide) which impacts the surface of the material. The impact body goes through a stage of movement toward the surface and then passes through an impact phase where the testing surface deforms plastically and elastically. The elastic recovery of the tested surface causes the rebound of the indenter into the Leeb hardness tester. The velocity of the travel of the indenter is directionally proportional to the force of the indenter after the initial impact phase[28]. The Leeb hardness value (HL) is the ratio between rebound velocity and the impact velocity multiplied by 1000. The measurement of elasticity was calculated using the pulse echo method with a Proceq Punditlab+. We measured Young’s (or elastic) modulus and attributed it to the general elasticity of the rock being measured (without separately measuring a modulus of shear strength or rigidity)[29]. This property reflects the ability for a rock to return to deform under stress and return to its original shape. As such, we hypothesize that rocks that have high rock stiffness are likely to transfer energy without plastic deformation making them more likely to fracture consistently. The pulse-echo method measures the speed of the transmission of ultrasonic waves through a rock. Conversion of ultrasonic values to Young’s modulus values requires an understanding of rock density and the length of a rock specimen. The measurement of P- and S- wave values was averaged over three measurements to remove potential variation caused by variation in manual coupling of transducers to the samples. S- wave velocity was measured five times using varied pulse intervals and rotation of transducers to identify possible courses of error. Values were averaged and the specific values for the elastic modulus were calculated using Punditlink software[30].

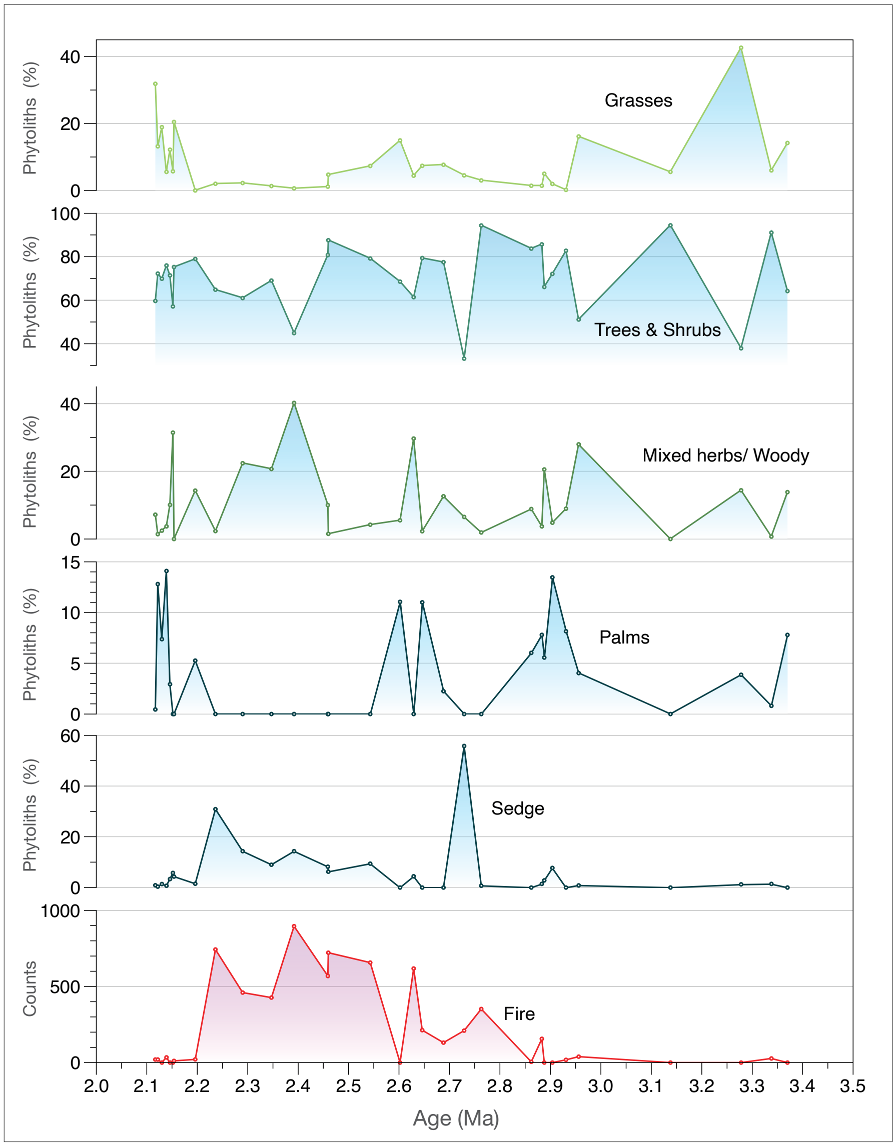

Supplementary Fig. 15 | Phytoliths - Samples collected from each of the defined stratigraphic boundaries (see Figure 2 for unit names). Phytolith counts are provided as percentages of total phytolith count. Note major changes in the grassland phytoliths after 2.6 Ma. In addition, we provide frequencies of microcharcoals through this sequence to provide an estimate of wildfire frequency. Four-fold increases in the prevalence of microcharcoals after 2.6 Ma indicate increase in drier habitats or an increase in fuel load, or both of these conditions. This is corroborated by the disappearance of palms (which require standing water). Similar shifts in pedogenic carbonate carbon isotopic signature suggest an increase in the proportions of open country habitats at this time[16] (Supplementary Figure 14).

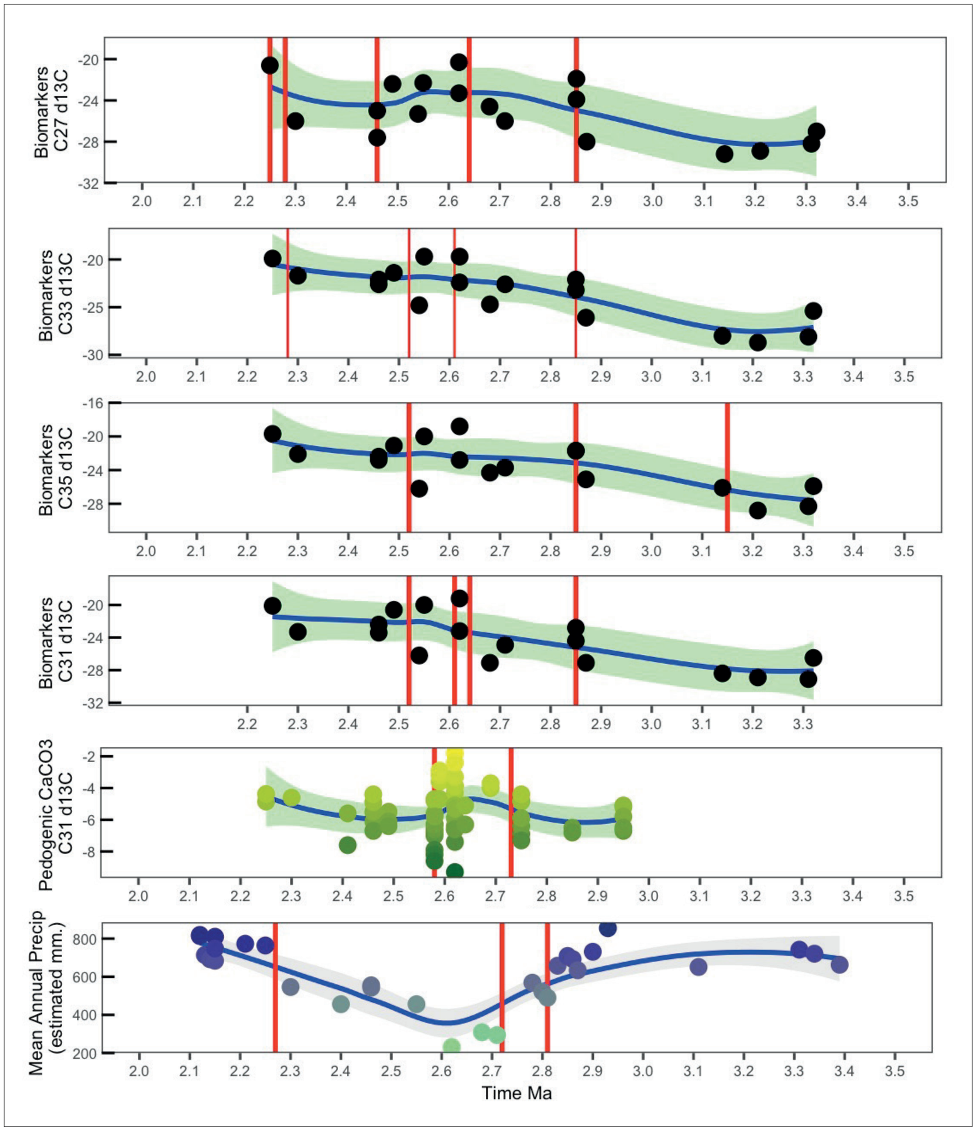

Supplementary Fig. 16 | Bayesian Change Point Analysis - We interrogate the time series analysis using a Bayesian change point analysis which can detect points in a time series where the statistical properties of the data change abruptly. As the Bayesian change point analysis uses the full range of variation in the sample, it is possible to identify shifts that are greater than would be expected by standard fluctuations in the data set. Bayesian change point algorithms use the exact posterior distribution of values to provide uncertainty bounds in the number and location of change points [31]. This type of analysis requires a value for each time step. We used linear interpolation at intervals of 30Ka between known data points (based on the age model estimates for each horizon in the stratigraphic sequence - linear interpolation with larger time intervals e.g. 50Ka -100 Ka produced indistinguishable results). Bayesian change points were calculated using a “burn in” value of 1000 which is used to insure model stability, we then used a 10000 step Markov Chain Monte Carlo iteration to identify the prior probabilities. Each proxy was run individually to assess the likelihood of changes in values through time representing a significant shift above expected ranges of variation in each paleoecological proxy. We use a .7 prior probability threshold to identify statistically significant changes in paleoecological proxies. Note that some proxies are limited by sedimentary context (e.g. pedogenic carbonates can only be found in soils) and thus are sometimes dispersed through the stratigraphic column. Note that most significant change points occur between 2.9 and 2.6 Ma suggesting an overall shift in ecological parameters at this time.

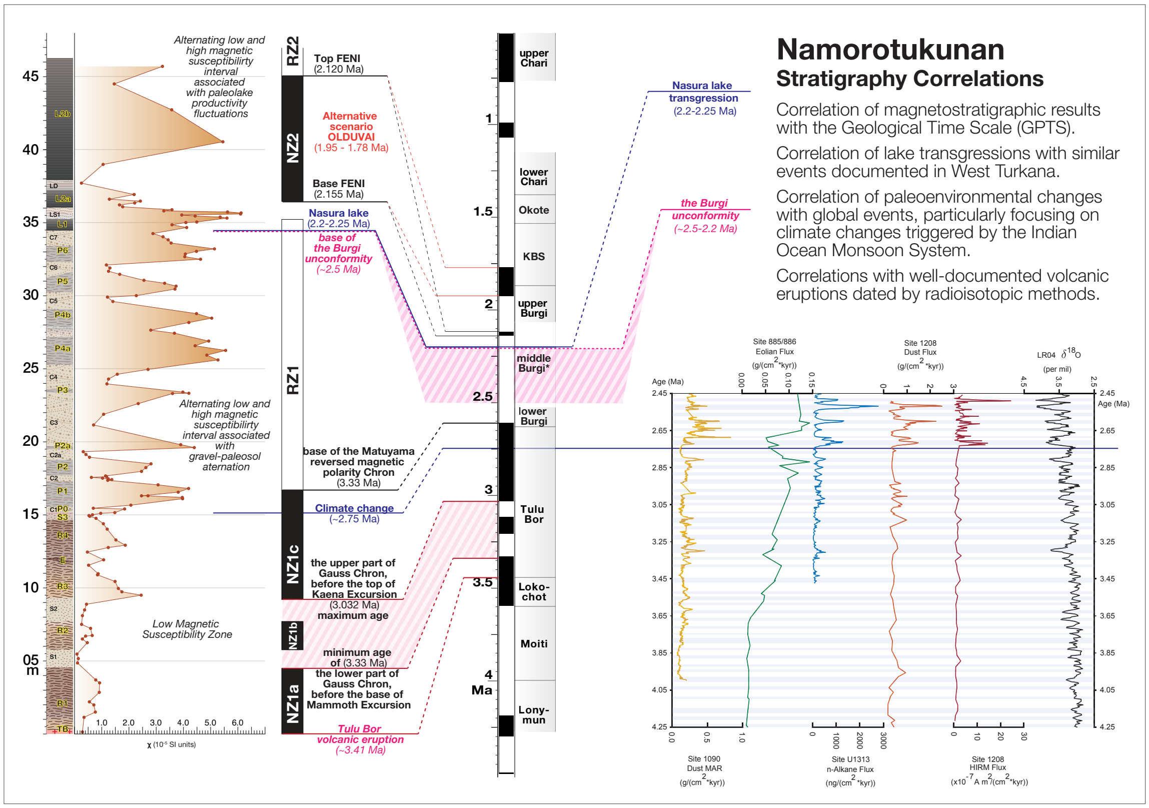

Supplementary Fig. 17 | Stratigraphic Correlations with Global Polarity Time Scale (GPTS). This figure illustrates the stratigraphic correlations based on four polarity zones identified in our studied interval aligned with the Global Polarity Time Scale (GPTS), a tephra level, a lake transgression and a climate shift, Key stratigraphic markers include: 1. A tephra at the base of our section, correlating with the 3.44 Ma Tulu Bor Tuff [32](McDougall and Brown, 2008), marking the lower boundary of our interval. 2. The uppermost unit correlates with the Lorenyang transgression of Lake Turkana (~2.1 Ma; [33–35]), providing an upper age limit. 3. Between these markers, the Gauss-Matuyama boundary (2.61 Ma; [36]) corresponds to the NZ1c-RZ1 boundary, suggesting the NZ2 interval aligns with the Feni paleomagnetic excursion. 4. The top of NZ1c is correlated with a climatic shift around 2.75 Ma. Due to incomplete records in the NZ1 interval, the Mammoth and Kaena magnetic subchrons are unlocated, with sedimentation rates suggesting a stratigraphic gap, likely between NZ1a and NZ1b. Two age models are developed (see Fig 4), with one accounting for the Burgi Unconformity (~2.5 Ma to ~2.2 Ma) in the upper part of RZ1.

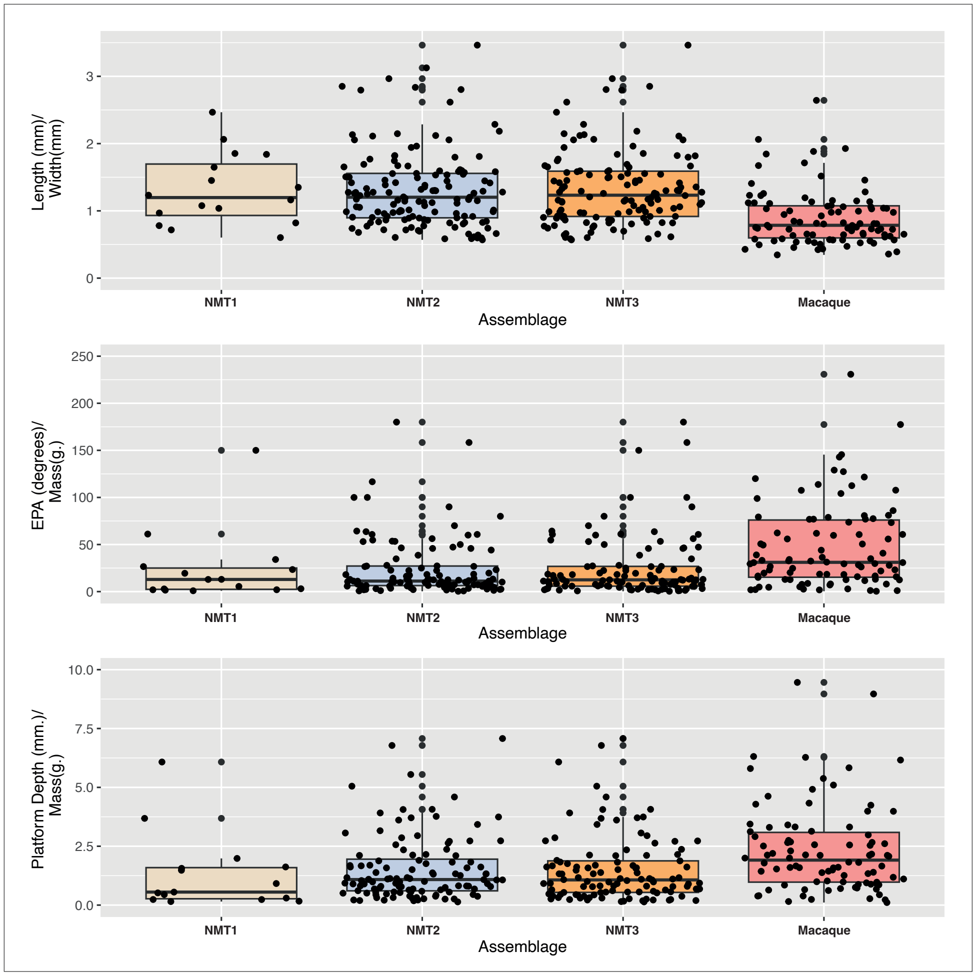

Supplementary Fig. 18 | Comparison with Macaque Flakes - Whole flakes from the three Namorotukunan collections are compared in technological attributes with the assemblage from Lobi Bay[37]. Box plots represent the interquartile range of values. The line in the center of the boxplot represents the median value. Whiskers on boxplots represent the first and third quartile plus (or minus) 1.5 times the interquartile range. There is significant overlap between the technological attributes in the Lobi Bay assemblage and that of the Namorotukunan assemblage. However, on an assemblage basis there are differences. Flakes produced by macaques tend to be wider relative to their length (Dunn's Test of multiple comparisons NMT1 vs. Macaque $p<.001$; NMT2 vs. Macaque $p<.001$; NMT3 vs. Macaque $p<.001$[38]). In addition, flakes produced by hominins at Namorotukunan tend to have higher EPA (External platform angle) values for flakes of similar mass. This suggests that hominins understood some of the relationship between EPA and mass of flakes (Dunn's Test of multiple comparisons NMT1 vs. Macaque $p<.05$; NMT2 vs. Macaque $p<.001$; NMT3 vs. Macaque $p<.001$). The hominins that produced the Namorotukunan assemblage also appear to understand the interaction between platform depth and mass. Hominins tend to have thicker platforms for flakes of similar mass. It is well known that platform depth, mass and external platform angle interact to impact the shape of flakes [39]. (Dunn's Test of multiple comparisons NMT1 vs. Macaque $p<.05$; NMT2 vs. Macaque $p<.05$; NMT3 vs. Macaque $p<.001$). All statistical comparisons used Holm's correction of p values to correct for multiple testing.

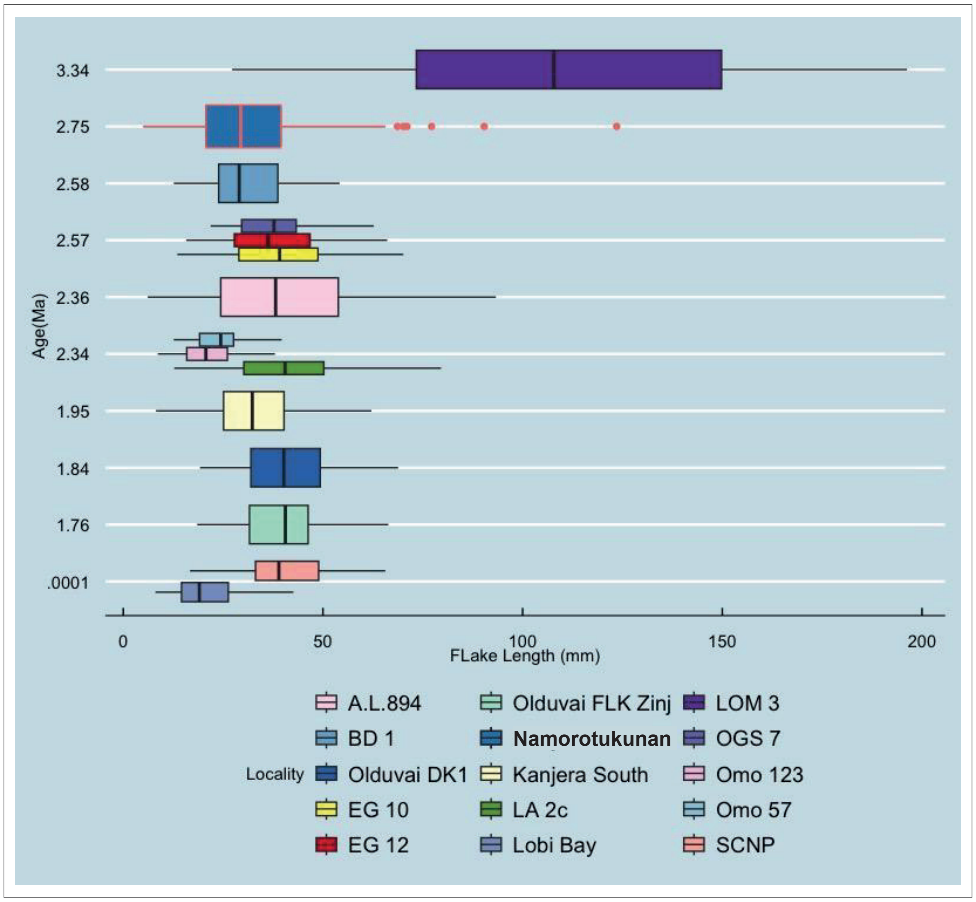

Supplementary Fig. 19 | Comparison with Oldowan Assemblages - Whole flakes from various Oldowan assemblages measured in mm. Boxplots represent the interquartile range of values. Whiskers at the end of boxplots represent the first and third quartile plus (or minus) 1.5 times the interquartile range. The line in the center of the boxplot represents the median value. Namorotukunan artifacts are highlighted in red. Note that the Namorotukunan assemblage is similar in size to BD1 (p=0.65) however the flakes from Namorotukunan are significantly smaller than all other Oldowan assemblages (with the exception of Kanjera South: p=0.26).

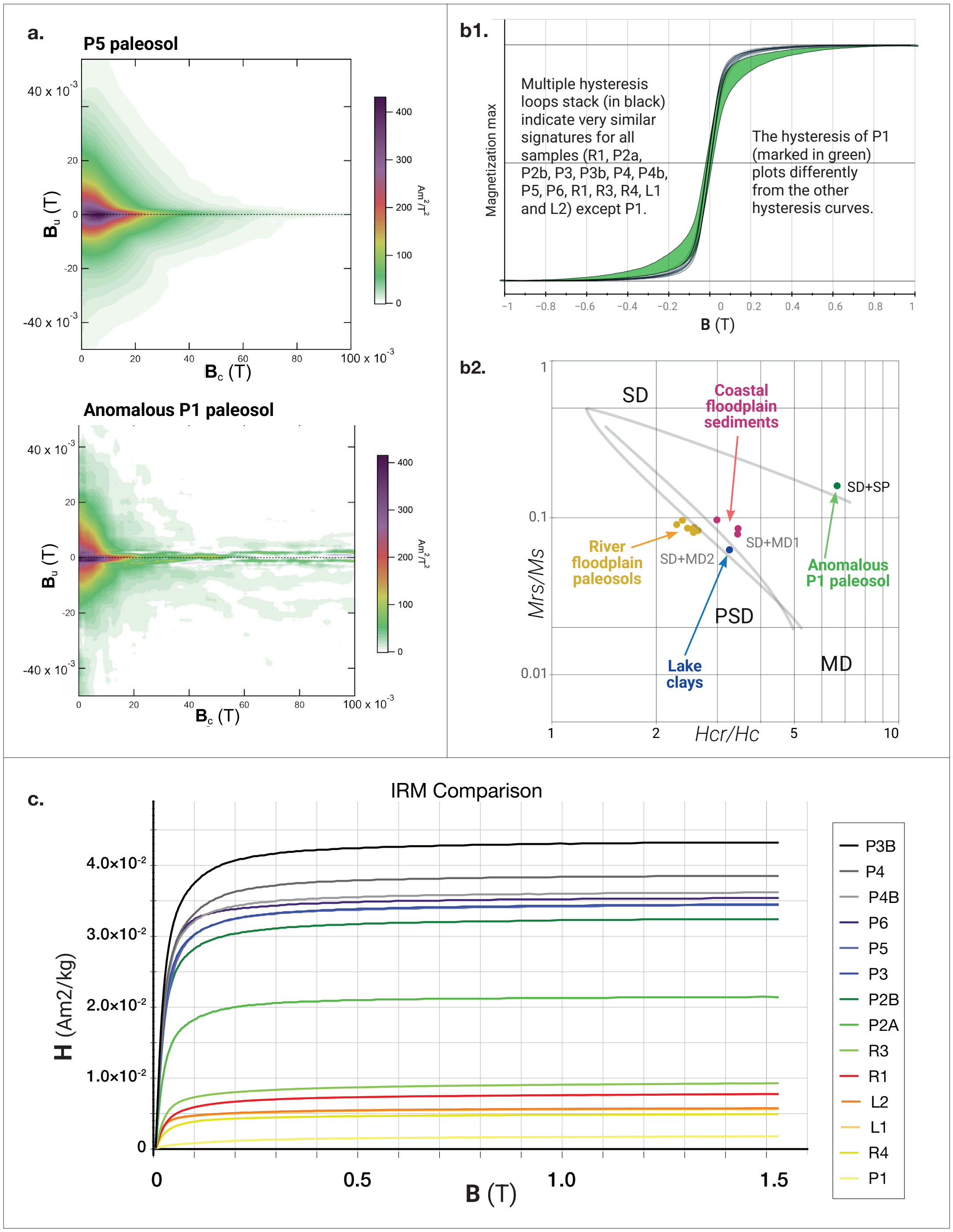

Supplementary Fig. 20 | Rock-magnetic results highlighting an unusual magnetic signature at the transition level. Detailed rock-magnetic analyses reveal a distinctive magnetic signature in sample P1, collected from the transition between coastal floodplains (samples R1–R4) and paleoriver systems (samples P1–P6). (a) First-Order Reversal Curve (FORC) diagrams generated using the FORCinel – NanoPaleoMagnetism software package, indicate poor preservation of magnetite in the paleosol level (P1) compared to sample P5. (b1) Hysteresis loops show that sample P1 contains a multicomponent magnetic carrier, contrasting with other samples. (b2) Day plots place sample P1 within the multi-domain (MD) region, whereas other samples cluster in the pseudo-single domain (PSD) region. Isothermal remanent magnetization (IRM) data further corroborate these findings, suggesting significant differences in magnetic mineralogy and domain states between samples.

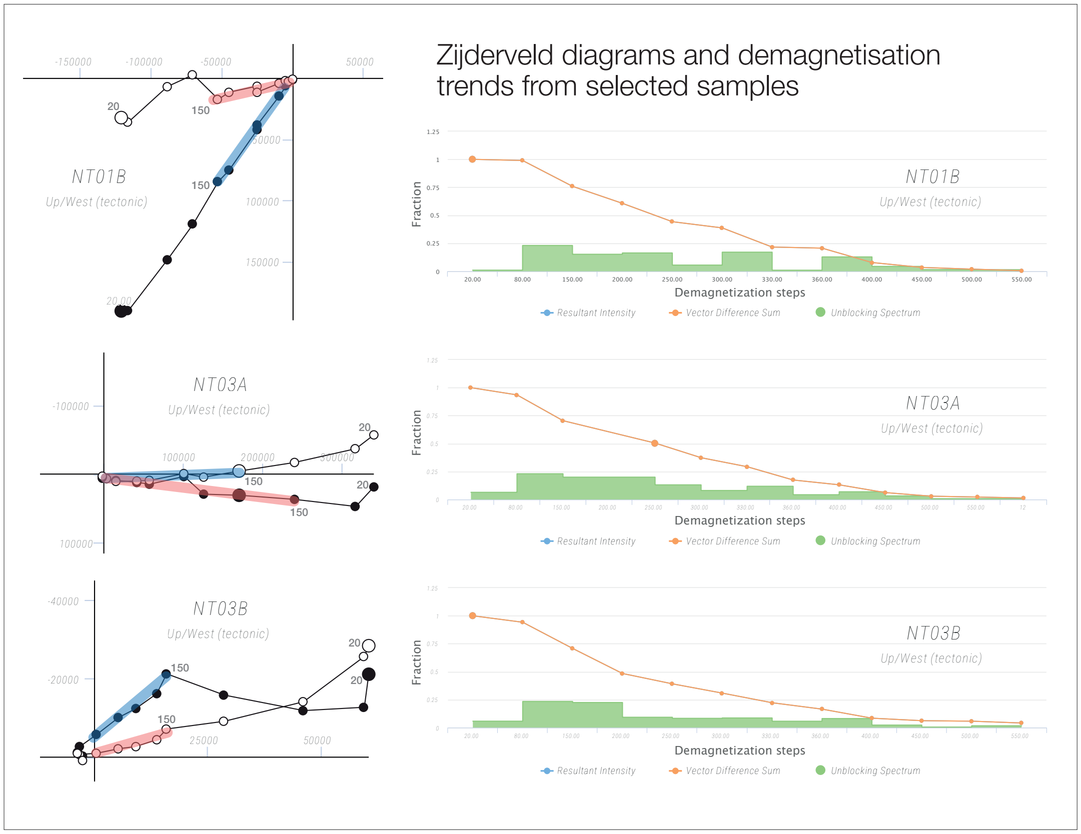

Supplementary Fig. 21 | Demagnetization trends. Zijderveld diagrams (right) show good signal preservation with indications of clockwise rotations (NT section). The blue and pink highlights indicate the measurement intervals used for calculating paleomagnetic directions. The magnetization/temperature plots (right) indicate near-complete demagnetization of the samples before reaching 600°C. All plots were generated using the Paleomagnetism.org platform, facilitating standardized analysis and interpretation of paleomagnetic data.

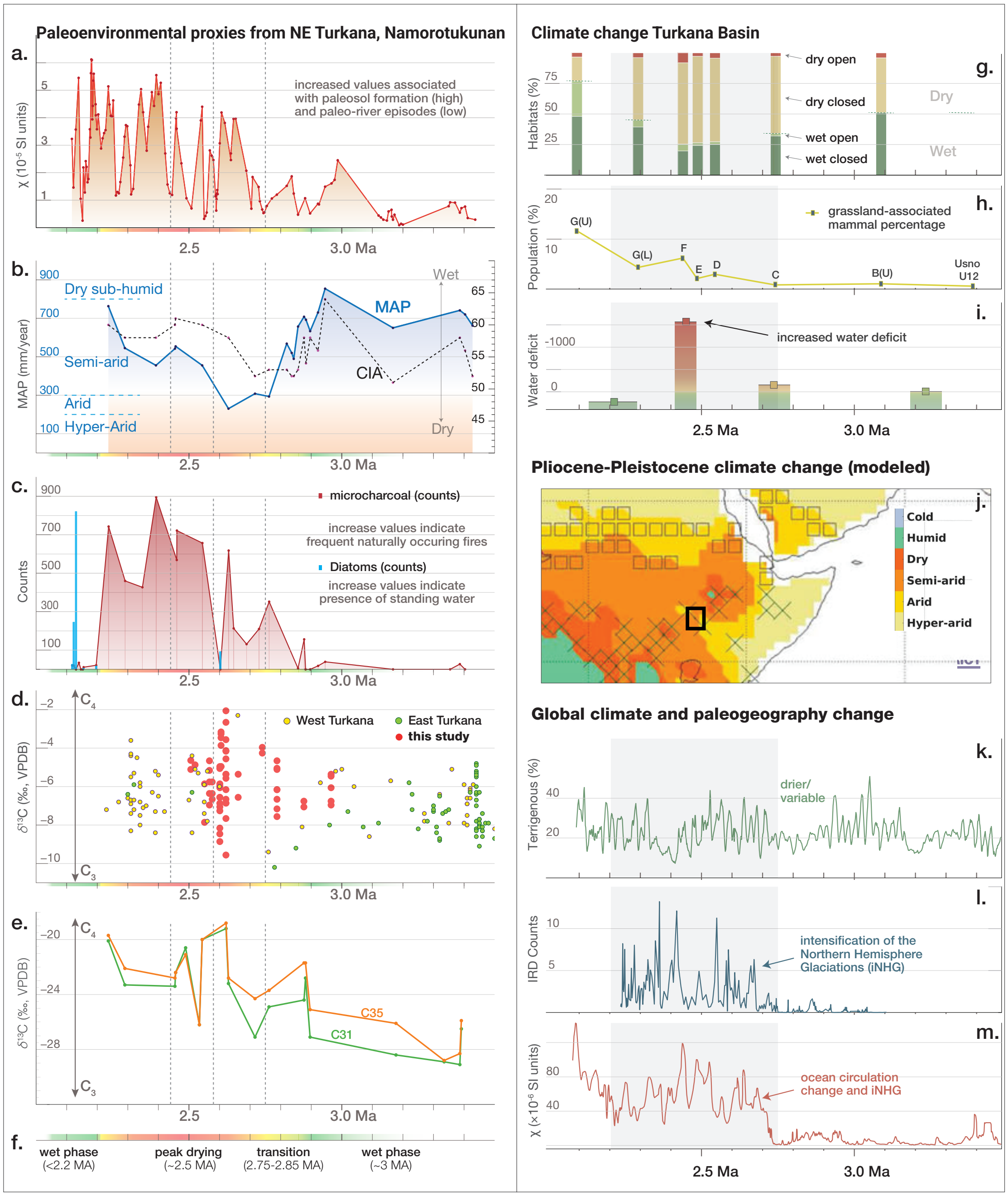

Supplementary Fig. 22 | Paleoenvironmental reconstructions.

(Local Scale) Paleoenvironmental change, based on proxy data obtained in this study: a. Magnetic susceptibility measured *in situ* in the Namorotukunan; b. Mean annual precipitation (MAP) estimates obtained from paleosol geochemistry (mm/year) follow a similar trend with the Chemical index of alteration (CIA), red numbered dots represent the three archaeological horizons (1. NMT1, 2. NMT2, 3. NMT3,); c. Microcharcoal and diatoms found in the phytolith samples (counts); d. Composite record of pedogenic carbonate $\delta^{13}C_{pedcarb}$ values from East and West Turkana, compiled from existing literature by Levin (2013) and combined with new paleosol $\delta^{13}C_{pedcarb}$ data from site Namorotukunan and its surroundings (‰, VPDB); e. Plant wax $\delta^{13}C$ values for odd-numbered *n*-alkane homologues C27 to C35; f. An overview of paleoclimatic trends observed in Namorotukunan, in this study.

(Basin Scale) Climate change in the Turkana Basin: g. Changes in the landscape composition based on faunal composition[40]; h. Changes in the abundance of open country adapted bovids [41]; i. Water deficit (ES-EI index) estimates (mm/year) based on $\delta^{18}O_{enamel}$ [42,43]).

(Regional Scale) East Africa j. Plio-Pleistocene climate trends modeled for East Africa: Change in the aridity pattern in the AWI-ESM low-CO2 experiment compared to the high-CO2 experiment colors indicate aridity according to the Aridity Index (after Zhong et al.[44]).

(Global Scale) Global climate change and paleogeographic changes during the Plio-Pleistocene: k. Terrigenous dust records in the Arabian Sea (%) from ODP 967 [45]; l. Ice-Rafted Debris (IRD) in the North Atlantic from ODP 907 [(counts), after Bartoli et al.[46]]; m. Magnetic Susceptibility in the North Pacific (ODP 882), after (Maslin and colleagues[47]);

Supplementary Tables

Supplementary Table 1: Geochemical characterization of the Tulu Bor Tuff in various parts of the Koobi Fora Formation as well as samples collected from Area 40. Samples of the Tulu Bor Tuff were collected at (4.399161N / 36. 326657 E WGS 84) and were based on previous descriptions of the Tulu Bor Tuff in Area 40[48]. Samples of the Tulu Bor Tuff were analyzed for geochemistry on a JEOL 8900 electron microprobe (EMP) instrument housed at the Carnegie Institution for Science's Geophysical Laboratory. Samples were measured using wavelength-dispersive x-ray spectroscopy (WDS) using the following analytical setup: accelerating voltage = 12 keV, beam current = 10 nA, and a beam diameter = 10 µm.

Sample	Analytical Conditions (keV, Na, SS)	**SiO2**	**TiO2**	**Al2O3**	**Fe2O3 /FeO**	**MnO**	**MgO**	**CaO**	**Na2O**	K2O	Total	**OT**	Age /Method	Reference
Tulu Bor B	20, 90nA, 1	76.61	0.19	12.60	1.88	0.03	0.03	0.22	4.59	3.85	100.00	91.89	3.40 Ma	Walter and Aronson (1993)[49]
K82-869	15, .01mA, 2	76.96	0.14	12.77	1.60	0.05	0.05	0.30	4.46	3.67	100.00	91.5	~3.3-3.4 Ma	Sarna-Wojcicki et al. (1985)[50]
K82-869	15, .1 mA, 58	76.98	0.14	12.78	1.60	0.04	0.05	0.29	4.46	3.64	100.00	91.65	3.3-3.4 Ma	Brown et al. (1992)[51]
TBB K81-529	20, 150pA, 1	76.71	0.17	12.78	1.69	0.03	0.03	0.25	4.63	3.70	99.99	100.0	3.4 Ma	Hart et al. (1992)[52]
DP-ET-14/T4	12, 5, 10	77.52	0.19	12.88	1.74	0.05	0.06	0.34	4.28	2.94	100.00	90.5		*This study*

Supplementary Table 2 | Lithological observations in the study area

Nr crt.	BEDS (lithological units)	DESCRIPTION AND OBSERVATIONS
**RED UNIT - Paleosols and lake floodplain sands**		
1	**TBT**	Tephra level previously described as corresponding to the Tulu Bor tuff
2	**R1**	Brown sandy silt with weakly developed paleosols
3	**S1**	Fine sands
4	**R2**	Reddish-brown silts with calcareous nodules
5	**S2**	Sand gravels with petrified wood (palm trees)
6	**R3**	Reddish-brown sands and clay, with poorly developed paleosol, weak pedogenic structure and black and red sandy intervals
7	**E**	Three or more discontinuous marker horizons consisting in well-cemented, reddish sandy silt and cobbles - groundwater/phreatic carbonate horizon
8	**R4**	Reddish sands with clay with root clasts and large rounded carbonate concretions
9	**S3**	Dark-red brownish clayey sands, with coarse red and black sand lenses and large rounded boulders at the base. On some boulders, weak oncoids are found.
**YELLOW UNIT - Paleosols and lake floodplain sands**		
10	**C1, C1a**	White yellowish sands and gravels, with fining upwards, imbrication and through bedding.
11	**P0**	Thin discontinuous black paleosol lenses, with elongated, vertical carbonate concretions (plant rhizomes and roots)
12	**P1**	Thick black paleosol with elongated, vertical carbonate concretions (plant rhizomes and roots). In the upper half of the paleosol sediments become a dark gleyed vertic argillisol with conchoidal features and slickensides
13	**C2**	White yellowish sands and gravels, with fining upwards, imbrication and through bedding.
14	**P2**	Black paleosol with elongated, vertical carbonate concretions (plant rhizomes and roots).
15	**C2a**	White yellowish sands and gravels, with fining upwards, imbrication and through bedding.
16	**P2a**	Thin discontinuous black paleosol lenses, with elongated, vertical carbonate concretions (plant rhizomes and roots)
17	**C3**	White yellowish sands and gravels, with fining upwards, imbrication and through bedding.
18	**P3**	Thick discontinuous black paleosol lenses, with elongated, vertical carbonate concretions (plant rhizomes and roots)
19	**C4**	Brown-reddish-yellowish sands and gravels, fining upwards, imbrication & through bedding.
20	**P4 a/b**	Thick brown sandy-silty paleosols with weak pedogenic structure and some sandy intervals
21	**C5**	White yellowish sands and gravels, with fining upwards, imbrication and through bedding.
22	**P5**	Brown sandy-silty paleosols with weak pedogenic structure. Large desiccation cracks, 20cm wide, 100cm long and at angles ≠120º filled with well-cemented sands from the beds above.
23	**C6**	White yellowish coarse sands and gravels, with fining upwards, imbrication and through bedding that are linked with a major river cut. The river direction can be followed in the landscape for a large distance (1-2 km) due to the sinkholes in the above-laying sediments.
24	**P6**	Brown sandy-silty paleosols with weak pedogenic structure and some sandy intervals
25	**C7**	White yellowish sands, ranging from loose to well cemented to the top
**Dark Grey unit - Lake clays with diatom-rich levels and lake sands**		
26	**L1**	Grey-black clays with horizontal bedding (lake offshore).
27	**LS1**	Yellow-white fine sands with rich catfish remains, well sorted, with horizontal bedding and in some levels wave ripples (upper shoreface).
28	**L2a**	Grey-black clays with horizontal bedding and fish remains (lake offshore).
29	**LS2**	Yellow-white fine sands, well sorted, with horizontal bedding and in some levels wave ripples, less developed than LS1 (upper shoreface).
30	**L2b**	Grey-black clays with horizontal bedding and diatom levels (lake offshore).

Supplementary Table 3 | Archaeological Finds at Each Archaeological Site

Site	Age	Core	Angular Fragment	Flake	Broken Flake	Percussion	Bone/Bone Fragment
NMT3	2.44 Ma	50	88	129	35	1	2
NMT2	2.60 Ma	65	484	160	35	2	15
NMT1	2.75 Ma	8	157	20	3	2	2

References

1. Harrison, R. J. & Feinberg, J. M. FORCinel: An improved algorithm for calculating first-order reversal curve distributions using locally weighted regression smoothing. *Geochemistry, Geophysics, Geosystems* **9**, (2008).
2. Egli, R. VARIFORC: An optimized protocol for calculating non-regular first-order reversal curve (FORC) diagrams. *Global and Planetary Change* **110**, 302–320 (2013).
3. Lepre, C. J., Margulies, G., Love, A., Ladapo, E. A. & Liutkus-Pierce, C. Additional evidence from Kenya to indicate a short reversal within the upper Olduvai Subchron. in vol. 2022 GP46A-06 (2022).
4. Mullender, T. A. *et al.* Automated paleomagnetic and rock magnetic data acquisition with an in-line horizontal "2 G" system. *Geochemistry, Geophysics, Geosystems* **17**, 3546–3559 (2016).
5. Nesbitt, Hw. & Young, G. Early Proterozoic climates and plate motions inferred from major element chemistry of lutites. *nature* **299**, 715–717 (1982).
6. Fedo, C. M., Wayne Nesbitt, H. & Young, G. M. Unraveling the effects of potassium metasomatism in sedimentary rocks and paleosols, with implications for paleoweathering conditions and provenance. *Geology* **23**, 921–924 (1995).
7. McLennan, S. M. Weathering and global denudation. *The Journal of Geology* **101**, 295–303 (1993).
8. Singh, M., Sharma, M. & Tobschall, H. J. Weathering of the Ganga alluvial plain, northern India: implications from fluvial geochemistry of the Gomati River. *Applied Geochemistry* **20**, 1–21 (2005).
9. Sheldon, N. D. & Tabor, N. J. Quantitative paleoenvironmental and paleoclimatic reconstruction using paleosols. *Earth-science reviews* **95**, 1–52 (2009).

10. Sheldon, N. D., Retallack, G. J. & Tanaka, S. Geochemical climofunctions from North American soils and application to paleosols across the Eocene-Oligocene boundary in Oregon. *The Journal of geology* **110**, 687–696 (2002).

11. Maynard, J. Chemistry of modern soils as a guide to interpreting Precambrian paleosols. *The Journal of Geology* **100**, 279–289 (1992).

12. Nordt, L. & Driese, S. New weathering index improves paleorainfall estimates from Vertisols. *Geology* **38**, 407–410 (2010).

13. Dzombak, R. M., Midttun, N. C., Stein, R. A. & Sheldon, N. D. Incorporating lateral variability and extent of paleosols into proxy uncertainty. *Palaeogeography, Palaeoclimatology, Palaeoecology* **582**, 110641 (2021).

14. Prochnow, S. J., Nordt, L. C., Atchley, S. C. & Hudec, M. R. Multi-proxy paleosol evidence for middle and late Triassic climate trends in eastern Utah. *Palaeogeography, Palaeoclimatology, Palaeoecology* **232**, 53–72 (2006).

15. Fortelius, M. *et al.* An ecometric analysis of the fossil mammal record of the Turkana Basin. *Philosophical Transactions of the Royal Society B: Biological Sciences* **371**, 20150232 (2016).

16. Wynn, J. G. Influence of Plio-Pleistocene aridification on human evolution! Evidence from paleosols of the Turkana Basin, Kenya. *Am. J. Phys. Anthropol.* **123**, 106–118 (2004).

17. Fernandez, M. H. & Vrba, E. S. Plio-Pleistocene climatic change in the Turkana Basin (East Africa): Evidence from large mammal faunas. *J. Hum. Evol.* **50**, 595–626 (2006).

18. Schick, K. D. *Stone Age Sites in the Making: Experiments in the Formation and Transformation of Archaeological Occurrences*. vol. 319 (British Archaeological Reports Ltd, 1986).

19. McPherron, S. P. Additional statistical and graphical methods for analyzing site formation processes using artifact orientations. *PLOS ONE* **13**, e0190195 (2018).

20. McPherron, S. J. Artifact orientations and site formation processes from total station proveniences. *Journal of Archaeological Science* **32**, 1003–1014 (2005).
21. Selvaggio, M. M. Carnivore Tooth Marks and Stone Tool Butchery Marks on Scavenged Bones - Archaeological Implications. *J. Hum. Evol.* **27**, 215–228 (1994).
22. Merritt, S. Factors affecting Early Stone Age cut mark cross-sectional size: implications from actualistic butchery trials. *J. Archaeol. Sci.* (2012).
23. Archer, W. *et al.* A geometric morphometric relationship predicts stone flake shape and size variability. *Archaeological and Anthropological Sciences* **10**, 1991–2003 (2018).
24. Dibble, H. L. & Rezek, Z. Introducing a new experimental design for controlled studies of flake formation: results for exterior platform angle, platform depth, angle of blow, velocity, and force. *Journal of Archaeological Science* **36**, 1945–1954 (2009).
25. Braun, D. R. *et al.* Earliest known Oldowan artifacts at> 2.58 Ma from Ledi-Geraru, Ethiopia, highlight early technological diversity. *Proceedings of the National Academy of Sciences* **116**, 11712–11717 (2019).
26. Proffitt, T. *et al.* Three-dimensional surface morphometry differentiates behaviour on primate percussive stone tools. *Journal of the Royal Society Interface* **18**, 20210576 (2021).
27. Harmand, S. Economic behaviors and cognitive capacities of early hominins between 2.34 ma and 0.70 Ma in West Turkana. *Mitteilungen der Gesellschaft fir Urgeschichte* **16**, 11–23 (2007).
28. Guan, S., Cao, R., Zhong, Y., Nan, H. & Wu, F. Comparison and combination of Leeb hardness and point load strength for indirect measuring tensile and compressive strength of rocks. *Bulletin of Engineering Geology and the Environment* **83**, 109 (2024).
29. Fort, R., Alvarez de Buergo, M., Perez-Monserrat, E. & Varas, M. J. Characterisation of monzogranitic batholiths as a supply source for heritage construction in the northwest of Madrid. *Engineering Geology* **115**, 149–157 (2010).

30. Wong, L. N. Y. & Maruvanchery, V. Different lithological varieties of Bukit Timah granite in Singapore: a preliminary comparison study on engineering properties. *Rock Mechanics and Rock Engineering* **49**, 2923–2935 (2016).

31. Ruggieri, E. & Antonellis, M. An exact approach to Bayesian sequential change point detection. *Computational Statistics & Data Analysis* **97**, 71–86 (2016).

32. McDOUGALL, I. & Brown, F. H. Geochronology of the pre-KBS Tuff sequence, Omo Group, Turkana Basin. *JGS* **165**, 549–562 (2008).

33. Nutz, A. *et al.* Plio-Pleistocene sedimentation in West Turkana (Turkana Depression, Kenya, East African Rift System): Paleolake fluctuations, paleolandscapes and controlling factors. *Earth-Science Reviews* **211**, 103415 (2020).

34. Boës, X. *et al.* Lake-level changes and hominin occupations in the arid Turkana basin during volcanic closure of the Omo River outflows to the Indian Ocean. *Quaternary Research* **91**, 892–909 (2019).

35. Lepre, C. J. Early Pleistocene lake formation and hominin origins in the Turkana–Omo rift. *Quaternary Science Reviews* **102**, 181–191 (2014).

36. Raffi, I. *et al.* The neogene period. in *Geologic time scale 2020* 1141–1215 (Elsevier, 2020).

37. Proffitt, T., Reeves, J. S., Braun, D. R., Malaivijitnond, S. & Luncz, L. V. Wild macaques challenge the origin of intentional tool production. *Science Advances* **9**, eade8159 (2023).

38. Dunn, O. J. Multiple Comparisons Using Rank Sums. *Technometrics* **6**, 241–252 (1964).

39. Li, L., Reeves, J. S., Lin, S. C., Tennie, C. & McPherron, S. P. Quantifying knapping actions: a method for measuring the angle of blow on flakes. *Archaeol Anthropol Sci* **14**, 156 (2022).

40. Negash, E. W. *et al.* Dietary trends in herbivores from the Shungura Formation, southwestern Ethiopia. *Proceedings of the National Academy of Sciences* **117**, 21921–21927 (2020).

41. Bobe, R., Behrensmeyer, A. K., Eck, G. G. & Harris, J. M. Patterns of abundance and diversity in late Cenozoic bovids from the Turkana and Hadar Basins, Kenya and Ethiopia Hominin Environments in the East African Pliocene: An Assessment of the Faunal Evidence. in (eds. Bobe, R., Alemseged, Z. & Behrensmeyer, A. K.) 129–157 (Springer Netherlands, 2007).

42. Blumenthal, S. A. *et al.* Aridity and hominin environments. *Proc. Natl. Acad. Sci. U.S.A.* **114**, 7331–7336 (2017).

43. Paquette, J. & Drapeau, M. S. M. Environmental comparisons of the Awash Valley, Turkana Basin and lower Omo Valley from upper Miocene to Holocene as assessed from stable carbon and oxygen isotopes of mammalian enamel. *Palaeogeography, Palaeoclimatology, Palaeoecology* **562**, 110099 (2021).

44. Zhong, Y. *et al.* Humidification of Central Asia and equatorward shifts of westerly winds since the late Pliocene. *Communications Earth & Environment* **3**, 274 (2022).

45. Larrasoaña, J. C., Roberts, A. P., Rohling, E. J., Winklhofer, M. & Wehausen, R. Three million years of monsoon variability over the northern Sahara. *Climate Dynamics* **21**, 689–698 (2003).

46. Bartoli, G. *et al.* Final closure of Panama and the onset of northern hemisphere glaciation. *Earth and Planetary Science Letters* **237**, 33–44 (2005).

47. Maslin, M. A. *et al.* 21. Northwest pacific site 882: The initiation of Northern Hemisphere Glaciation. in *Proceedings of the Ocean Drilling Program* vol. 145 179–94 (1995).

48. Kidney, C. L. *Pliocene Stratigraphy and Geology of the Northeastern Ileret Region, Kenya*. (The University of Utah, 2012).

49. Walter, R. C. & Aronson, J. L. Age and source of the Sidi Hakoma tuff, Hadar formation, Ethiopia. *Journal of Human Evolution* **25**, 229–240 (1993).

50. Sarna-Wojcicki, A. M., Meyer, C. E., Roth, P. H. & Brown, F. H. Ages of tuff beds at East African early hominid sites and sediments in the Gulf of Aden. *Nature* **313**, 306–308 (1985).

51. Brown, F. H. Tulu Bor Tuff at Koobi Fora correlated with the Sidi Hakoma Tuff at Hadar. in *Nature* vol. 300, nos. 5893, 1982 631–633, ill (London, 1982).

52. Hart, W. K., Walter, R. C. & WoldeGabriel, G. Tephra sources and correlations in Ethiopia: application of elemental and neodymium isotope data. *Quaternary International* **13**, 77–86 (1992).
