# Supplementary material for: Early Oldowan technology thrived during Pliocene environmental change in the Turkana Basin, Kenya
Source: Nat Commun. 2025 Nov 4;16:9401. doi: 10.1038/s41467-025-64244-x (PMC12586688; doi:10.1038/s41467-025-64244-x)
Supplement: Supplementary file 10 — Reporting Summary [file 41467_2025_64244_MOESM10_ESM.pdf]

nature portfolio

Corresponding author(s): David R. Braun, Dan V. Palcu

Last updated by author(s): Jul 9, 2024

Reporting Summary

Nature Portfolio wishes to improve the reproducibility of the work that we publish. This form provides structure for consistency and transparency in reporting. For further information on Nature Portfolio policies, see our Editorial Policies and the Editorial Policy Checklist.

Statistics

For all statistical analyses, confirm that the following items are present in the figure legend, table legend, main text, or Methods section.

n/a	Confirmed	
☐	☒	The exact sample size (*n*) for each experimental group/condition, given as a discrete number and unit of measurement
☐	☒	A statement on whether measurements were taken from distinct samples or whether the same sample was measured repeatedly
☐	☒	The statistical test(s) used AND whether they are one- or two-sided<br>*Only common tests should be described solely by name; describe more complex techniques in the Methods section.*
☐	☒	A description of all covariates tested
☐	☒	A description of any assumptions or corrections, such as tests of normality and adjustment for multiple comparisons
☐	☒	A full description of the statistical parameters including central tendency (e.g. means) or other basic estimates (e.g. regression coefficient) AND variation (e.g. standard deviation) or associated estimates of uncertainty (e.g. confidence intervals)
☐	☒	For null hypothesis testing, the test statistic (e.g. *F*, *t*, *r*) with confidence intervals, effect sizes, degrees of freedom and *P* value noted<br>*Give P values as exact values whenever suitable.*
☒	☐	For Bayesian analysis, information on the choice of priors and Markov chain Monte Carlo settings
☒	☐	For hierarchical and complex designs, identification of the appropriate level for tests and full reporting of outcomes
☒	☐	Estimates of effect sizes (e.g. Cohen's *d*, Pearson's *r*), indicating how they were calculated

Our web collection on statistics for biologists contains articles on many of the points above.

Software and code

Policy information about availability of computer code

Data collection	Archaeological data was collected using open source data entry software available at OldStoneAge.com
Data analysis	Most data analysis was conducted in R (Version 4.0) when specific packages were used for specific analyses these are mentioned in the text and supplementary information.

For manuscripts utilizing custom algorithms or software that are central to the research but not yet described in published literature, software must be made available to editors and reviewers. We strongly encourage code deposition in a community repository (e.g. GitHub). See the Nature Portfolio guidelines for submitting code & software for further information.

Data

Policy information about availability of data

All manuscripts must include a data availability statement. This statement should provide the following information, where applicable:

- Accession codes, unique identifiers, or web links for publicly available datasets
- A description of any restrictions on data availability
- For clinical datasets or third party data, please ensure that the statement adheres to our policy

Artifacts and fossils collected are housed at the National Museums of Kenya. Sample numbers refer to accession numbers described in National Museums of Kenya databases. Much of the data in the analysis is available in attached .csv and .xls files. Other data is available upon request

Research involving human participants, their data, or biological material

Policy information about studies with human participants or human data. See also policy information about sex, gender (identity/presentation), and sexual orientation and race, ethnicity and racism.

Reporting on sex and gender	No human subjects data was collected in this study
Reporting on race, ethnicity, or other socially relevant groupings	No human subjects data was collected in this study
Population characteristics	No human subjects data was collected in this study
Recruitment	No human subjects data was collected in this study
Ethics oversight	No human subjects data was collected in this study

Note that full information on the approval of the study protocol must also be provided in the manuscript.

Field-specific reporting

Please select the one below that is the best fit for your research. If you are not sure, read the appropriate sections before making your selection.

☐ Life sciences ☐ Behavioural & social sciences ☒ Ecological, evolutionary & environmental sciences

For a reference copy of the document with all sections, see nature.com/documents/nr-reporting-summary-flat.pdf

Ecological, evolutionary & environmental sciences study design

All studies must disclose on these points even when the disclosure is negative.

Study description	This study reports on new excavations and the associated environmental and contextual information in Pliocene sediments in Kenya. We provide detailed analysis of the contextual data (proxies of ancient habitats and age estimates) in the methods and supplementary data.
Research sample	The research sample includes excavated artefacts and fossils from the Koobi Fora Formation in northern Kenya and associated contextual and paleoenvironmental data. The sample includes materials collected from excavations, surface surveys and geological trenches.
Sampling strategy	The paleomagnetic and geochemistry sampling resolution was determined by the presence of suitable lithological units (e.g., mudstones, sandstones, paleosols) with a degree of lithification that allowed the drilling and retrieving of small, oriented cores (sampling parameters detailed in the methods).
Data collection	Lithic and faunal data from the excavated samples were collected using methods described in the manuscript. Comparative analaysis includes data collated from publications cited in the text. The paleomagnetic and geochemical sampling involved the collection of geographic coordinates and the measurement of sample & bedding orientation parameters for the paleomagnetic samples (these procedures are described in the methods section).
Timing and spatial scale	The excavated localities described here were identified in 2012. Continued field research extended over 13 years with successive excavations and stratigraphic sampling. Paleomagnetic and geochemical sampling was developed over a series of field seasons, at first in the early stages of the project to confirm the existing age interpretations (2012) and the later (in 2018, 2019 and 2022) stages to improve the existing age interpretations in resonance with the archaeological finds, reflecting the development of our geological models and stratigraphy interpretations.
Data exclusions	no data was excluded from the analysis
Reproducibility	We provide raw data for all environmental and geological analyses. Artifact analysis provides data for the proportion of rock types and measures used in comparison with other assemblages,
Randomization	Analysis of geochemical data included all samples so that no randomization of sampling procedure is required. Phytolith randomization procedures follow methods previously published for this technique and outlined in the methods section. Lithic samples were measured in their entirety. Paleomagnetic data was collected and analyzed in separate labs to insure independent assessment of results. We did not use a randomization procedure except for when we analyzed details of the core forms from three dimensional models, Here we used a permutation test that randomly samples from the entire data set 1000 times to create a probability of difference. This is a standard permutation technique used when sample sizes are low and data is not normally distributed.
Blinding	Blinding is not relevant to our study. We did not use blinding for any analysis.

Did the study involve field work? ☒ Yes ☐ No

Field work, collection and transport

Field conditions	Fieldwork was performed near Equator during the dry season (June-August) at high ambient temperatures (20-40ºC)
Location	All fieldwork was conducted in the Ileret Region of the Turkana Basin in the Koobi Fora Formation. Detailed maps with precise location of artifacts and fossils are provided in the main text and supplementary information.
Access & import/export	All samples were collected under the auspices (and in collaboration with) the National Museums of Kenya. Materials were exported using standard export procedures delineated by the National Museums of Kenya. Export permits were recieved for all samples that were transported out of Kenya.
Disturbance	Excavations resulted in relatively minimal footprint on the landscape in northern Kenya. Excavations were backfilled when appropriate (when the excavation represents a risk to people or livestock in the region).

Reporting for specific materials, systems and methods

We require information from authors about some types of materials, experimental systems and methods used in many studies. Here, indicate whether each material, system or method listed is relevant to your study. If you are not sure if a list item applies to your research, read the appropriate section before selecting a response.

Materials & experimental systems

n/a	Involved in the study	
☒	☐	Antibodies
☒	☐	Eukaryotic cell lines
☐	☒	Palaeontology and archaeology
☒	☐	Animals and other organisms
☒	☐	Clinical data
☒	☐	Dual use research of concern
☒	☐	Plants

Methods

n/a	Involved in the study	
☒	☐	ChIP-seq
☒	☐	Flow cytometry
☒	☐	MRI-based neuroimaging

Palaeontology and Archaeology

Specimen provenance	Fossils and artefacts (as well as geological samples) were collected with a total station or a DGPS system to ensure accurate location information to the nearest cm. Coordinates of all samples and artifacts are provided to the National Musuems of Kenya as part of the procedure of accession. Permits for research are provided by the National Council of Science and Technology. Permits were applied for during every year of field research. In addition we received a permit for exploration and excavation from the Ministry of Sports, Culture and Heritage (now known as the department of Culture, the Arts and Heritage).
Specimen deposition	All fossils and artifacts have been accessioned withe Archaeology Department of the National Museums of Kenya.
Dating methods	We provide age estimates based on age models calculated using paleomagentic and tephrostratigrpahic methods. The only radiometric age we report if the Tulu Bor Tuff which has been the subject of numerous previous radiometric age assessments. None of these age estimates require calibration.

☒ Tick this box to confirm that the raw and calibrated dates are available in the paper or in Supplementary Information.

Ethics oversight	All research conducted in Kenya is under the guidance of the National Museums of Kenya which is the sole repository of Kenya's heritage as per Kenyan law.

Note that full information on the approval of the study protocol must also be provided in the manuscript.

Plants

Seed stocks	N/A
Novel plant genotypes	N/a
Authentication	N/A
